# Bioinspired Tactile Sensation Based on Synergistic Microcrack-Bristle Structure Design toward High Mechanical Sensitivity and Direction-Resolving Capability

**DOI:** 10.34133/research.0172

**Published:** 2023-06-16

**Authors:** Yiqun Zhang, Qi Liu, Wenjuan Ren, Yangyang Song, Hua Luo, Yangyang Han, Liang He, Xiaodong Wu, Zhuqing Wang

**Affiliations:** ^1^ School of Mechanical Engineering, Sichuan University, Chengdu 610065, China.; ^2^ Med+X Center for Manufacturing, West China Hospital, Sichuan University, Chengdu 610041, China.; ^3^ State Key Laboratory of Polymer Materials Engineering, Polymer Research Institute of Sichuan University, Chengdu 610065, China.

## Abstract

Natural tactile sensation is complex, which involves not only contact force intensity detection but also the perception of the force direction, the surface texture, and other mechanical parameters. Nevertheless, the vast majority of the developed tactile sensors can only detect the normal force, but usually cannot resolve shear force or even distinguish the directions of the force. Here, we present a new paradigm of bioinspired tactile sensors for resolving both the intensity and the directions of mechanical stimulations via synergistic microcrack-bristle structure design and cross-shaped configuration engineering. The microcrack sensing structure gives high mechanical sensitivity to the tactile sensors, and the synergistic bristle structure further amplifies the sensitivity of the sensors. The cross-shaped configuration engineering of the synergistic microcrack-bristle structure further endows the tactile sensors with good capability to detect and distinguish the directions of the applied mechanical forces. The as-fabricated tactile sensors exhibit a high sensitivity (25.76 N^−1^), low detection limit (5.4 mN), desirable stability (over 2,500 cycles), and good capability to resolve both mechanical intensity and directional features. As promising application scenarios, surface texture recognition and biomimetic path explorations are successfully demonstrated with these tactile sensors. This newly proposed tactile sensation strategy and technology have great potential applications in ingenious tactile sensation and construction of various robotic and bionic prostheses with high operational dexterity.

## Introduction

Tactile sensation is an important way for creatures to acquire the outside information. For most animals, tactile sensory organs can be found all over their body, responsible for perceiving a variety of environmental stimulations, especially mechanical stimuli. To imitate natural tactile sensory organs, artificial tactile sensors have been developed as a class of devices or systems that can be used to obtain external information such as temperature [1–3], humidity [4,5], texture [6], normal force [2,7,8], and shear force [9,10]. As an indispensable component of the burgeoning smart wearables such as electronic skins [11], wearable devices [12,13], soft robots [14], and human–machine interfaces [15], tactile sensors with comparable or even superior sensing capabilities than natural human skin or animal sensory organs have attracted enormous attention from both academia and industry field.

In recent years, great progress has been made for tactile sensors, including novel structural engineering [16–18], sensing mechanism innovation [19–21], and development of new materials [22]. For example, a variety of microstructures, such as pyramids [23,24], hemispheres, and micropores, are used to replace planar structures to improve the sensitivity and resolution of sensors, which has become a research trend for tactile sensing devices [25]. For example, Yang et al. [26] designed an ultrasensitive capacitive mechanical sensor based on a porous pyramid dielectric layer structure. The sensor not only showed high sensitivity up to 44.5 kPa^−1^ in the pressure range of <100 Pa but also exhibited excellent temperature insensitivity and stretchability. In addition to the regular microstructures prepared by traditional photolithography processes, irregular structures found in nature, such as roses, lotus leaves, sandpaper, and silk, also provide researchers with a variety of inspirations [27,28]. Zhao et al. [29] reported a biomimetic tactile sensor with spinous process structure templated from the lotus leaf surface. The sensor with spinous process structure has a high sensitivity (507 kPa^−1^) and great linearity in the low-pressure region (0 to 5.57 kPa). In addition to sensor performance improvement, researchers have also proposed new tactile sensors by adopting new sensing mechanisms [19–21]. For instance, Kim et al. [30] demonstrated a soft, stretchable, and transparent surface-capacitive system based on polyacrylamide hydrogels containing lithium chloride salts, which can sense the touched position and perceive the writing of words. Besides, Chun et al. [31] prepared a self-powered mechanical sensor based on an artificial ion-channel system and piezoelectric film for dynamic and static stimulations. Combining these 2 sensing mechanisms, mechanical stress and surface roughness can be distinguished and detected. Despite the marked progress made as mentioned above, there is still a long way to go before realizing the full mimicking of tactile sensation functionality in humans or other animals. This is due to that natural tactile sensation not only involves force intensity detection but also includes the perception of other complex mechanical parameters such as force direction, contact location, surface texture, and so on. However, the vast majority of the developed tactile sensors are only capable of detecting the normal force but are usually unable to resolve shear force or even distinguish the directions of the force.

To resolve both normal force and shear force, recently, Lee et al. [32] demonstrated a flexible capacitive tactile sensor array with 8 × 8 sensing units. Each sensing unit consists of 4 capacitors, which decompose the contact force into normal and shear components. Its sensitivities in the *x*, *y*, and *z* directions are 2.5%/mN, 2.9%/mN, and 3.0%/mN, respectively. Besides, Zhang et al. [33] reported a piezoelectric tactile sensor based on a rigid-flexible hybrid force transmission layer in combination with a soft bottom substrate for detecting the direction of multiple forces. Experiments show that this sensor has a high sensitivity of 346.5 pC N^−1^ and a broad detection range of 0.009 to 4.3 N. In addition, by mimicking the interlocking dermis–epidermal interface in human skin, Boutry et al. [9] reported a capacitive biomimetic flexible electronic skin for real-time measurement and differentiation of normal and tangential forces. The e-skin exhibits high sensitivity (up to 0.19 kPa^−1^) and good cycling stability (3,000 cycles). However, these sensors mentioned above are all fabricated based on micro-electromechanical system (MEMS) technologies (e.g., photolithography, etching, and vacuum deposition), which are expensive and time-consuming and limit their large-area applications [9,32–35]. Moreover, most of the abovementioned sensors are based on capacitive and piezoelectric sensing mechanisms. Capacitive sensors usually suffer from parasitic capacitance and are susceptible to interference, while piezoelectric sensors are not suitable for static force detection [36]. In contrast, piezoresistive sensors are easy to fabricate and operate, not susceptible to interference, and can detect static forces [37]. Developing a new paradigm of piezoresistive tactile sensors that can resolve the complex parameters of mechanical stimulations (e.g., intensity and direction) based on scalable and cost-efficient fabrication methodologies has great significance but has rarely been demonstrated so far.

**Fig. 1.**
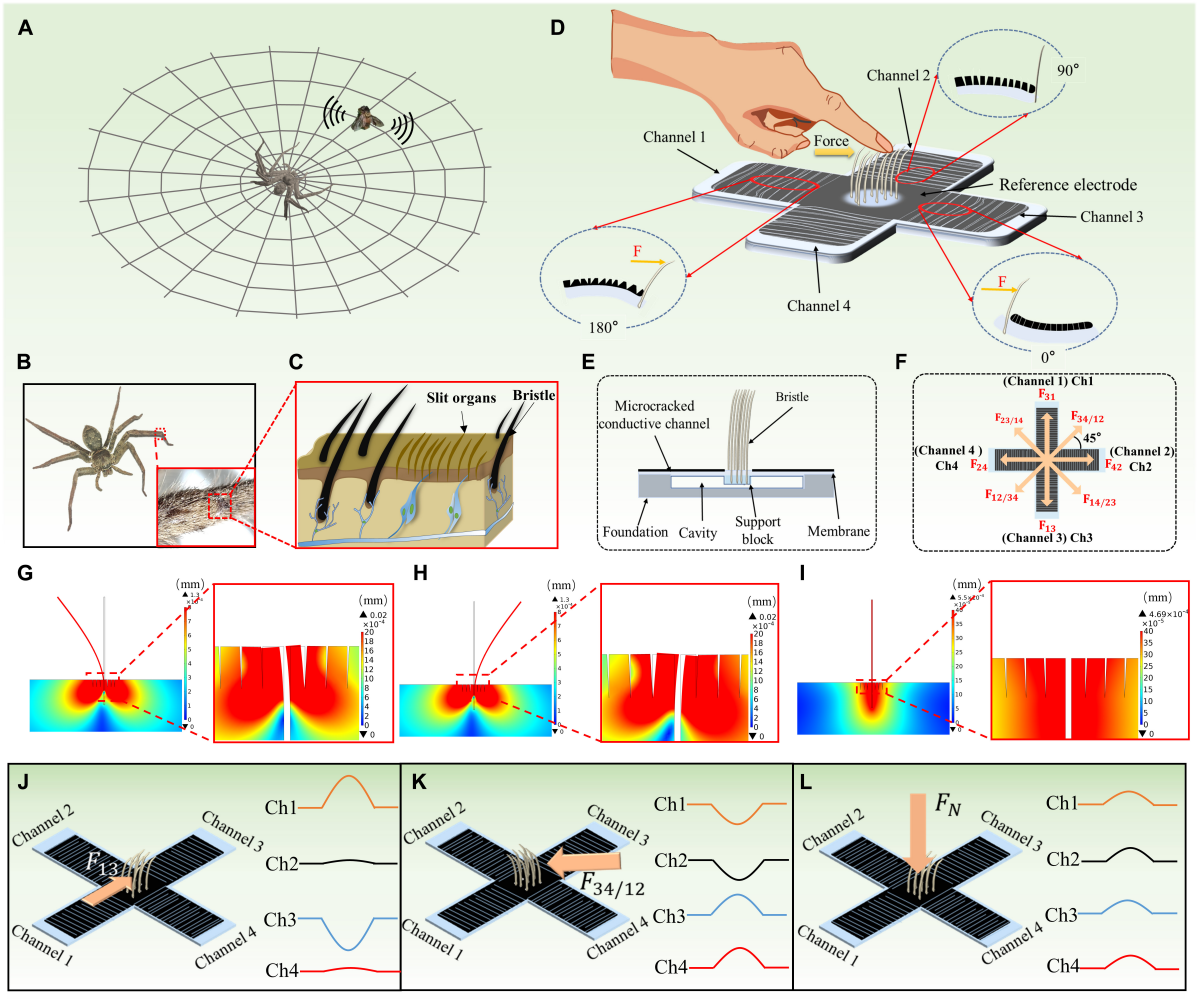
The design concept and operating principle of bioinspired tactile sensors. (A to C) Illustrations showing that spiders have highly sensitive organs for detecting the direction of external mechanical stimulus. The inset is an enlarged image of the leg joint between the metatarsal and tarsus. These slit organs and bristles are connected to the nervous system and can monitor tiny external forces and vibrations. (D) Schematic diagram depicting the structure layout and basic working principle of the tactile sensors. (E) Schematic giving the cross-section of the tactile sensors. (F) The top view of the tactile sensors and the schematic diagram showing the shear force directions (for example, the direction of shear force from channel 1 to channel 3 is labeled as *F*_13_, and the direction of shear force from channel 3/channel 4 to channel 1/channel 2 is labeled as *F*_34/12_). (G to I) Finite element analysis (FEA) of the microcrack-bristle synergetic structure when applying a shear force from right to left (G) and from left to right (H) and applying a normal force from the top of the structure (I). (J to L) Schematic diagram showing the typical signal outputs from the 4 channels when applying mechanical forces *F*_13_ (J) and *F*_34/12_ (K) and a normal force (L).

Spiders can perceive the directions of the trapped prey by detecting the mechanical stimuli generated by the prey's struggling on the web, as shown in Fig. 1A. Their ultrasensitive mechanical sensing capability benefits from the special sensory organs near their leg joints. As shown in Fig. 1B and C, in the sensory organs of spiders, there are 2 structures that contribute to their high mechanical sensitivity. One is the bristle structure on top of the mechanoreceptors, which resembles a cantilever beam structure and acts as a mechanical signal amplifier [38,39]. External mechanical stimulations, such as vibration, touch pressure, and flow of fluids, can cause the bending deformation of the spider's bristles. This causes the bristles to vibrate or deform and finally transmit the stimuli to the nervous system by pulling the dendrites of sensory neurons connected to them (Fig. 1C). In addition to the bristle structure near the receptors, another unique structure is the slit organs. Each slit organ is connected by a pair of neurons, called bipolar neurons [38,40]. Tiny external stimulations can easily cause the deformation of the slit organs, which transmits the signal to the bipolar neuron to detect the mechanical signals. Importantly, these 2 organs are extremely sensitive, which significantly improves the mechanical sensitivity of the spiders’ sensory organs and endows natural spiders with superior capability to sense and perceive the subtle and elusive mechanical stimulations in the surrounding environment.

Inspired by the slit organs and bristles of spiders, in this work, we propose and demonstrate a new class of highly sensitive piezoresistive tactile sensors that can well detect the intensity and distinguish the directions of mechanical stimulations. The realization of such sensor functionalities is enabled by the synergetic design and construction of 2 biomimetic structures: microcrack structure and bristle structure. The microcrack structure endows the sensors with high mechanical sensitivity [41,42], while the bristle structure further enhances the sensor sensitivity via a cantilever amplifying effect [39,43]. More importantly, the sensors feature good ability to detect the directions of the externally applied force via a piezoresistive sensing mechanism, which has rarely been explored but proved to be effective in this work. Via the synergistic microcrack-bristle structure design and the cross-shaped device configuration engineering, the as-prepared sensors exhibited a high sensitivity (25.76 N^−1^) when applying shear force and normal force. In addition, the tactile sensing has a low detection limit (5.4 mN) and good stability (over 2,500 cycles). Moreover, we demonstrated that the sensors exhibited desirable responses and good repeatability to both shear forces and normal force in different directions, verifying the direction-resolving capability of such sensors. The superiorities of the sensor based on the proposed synergistic microcrack-bristle structure design compared with other reported tactile sensors with direction-resolving capability are systematically presented in Fig. S1, Table S1, and Note S2. As proof-of-concept demonstrations, the fabricated tactile sensors were successfully applied in application scenarios including surface texture recognitions and biomimetic path explorations. These newly proposed tactile sensors have promising application prospects in wearable devices, soft medical robots, and biomimetic prostheses with high operating dexterity.

## Results

### Design concept and working mechanism of the bioinspired tactile sensors

The slit sensilla and hair-like structures of spiders are extremely sensitive to mechanical signals. Inspired by these 2 structures in spider sensory organs (Fig. 1C), here, we design a new paradigm of highly sensitive piezoresistive tactile sensors with unique ability to resolve not only the intensity but also the directions of the external mechanical stimulations. As shown in Fig. 1D, the tactile sensors consist of a signal amplifying bristle structure surrounded by 4 highly sensitive microcracked channels distributed in the 4 directions of the cross-shaped structure. Benefiting from the synergistic effect of the microcrack structure and the bristle structure, the tactile sensors have superior sensitivity than the sensors with only one structure design (as demonstrated below in Fig. 3, B to E). More importantly, the directions of the external mechanical stimulations applied at the tip of bristle structures can be well resolved by simultaneously recording and analyzing the response signal outputs from the 4 microcrack channels, which greatly enhance the functionality and enrich the application scenarios of such tactile sensors. To realize the synergistic effect of the microcrack-bristle structures, rational structure engineering and configuration design are necessary. For the structure design (Fig. 1E), when the bristle tip is stimulated by force, the cavity in the middle of the sensors can provide enough space for the deformation of the bristle root part, thus ensuring adequate disconnection–reconnection process of the microcrack structures in the 4 channels. The measuring electrodes of the sensors are located at the end of each channel. Note that the 4 channels share a single reference electrode located in the middle of the sensors, which surrounds the bristle region. Most importantly, since the 4 microcrack channels are distributed in 4 directions perpendicular to each other, the directions and intensity of the mechanical forces can be reflected by the change in resistances of each channel between the measuring electrodes and the reference electrode.

The working mechanism of the tactile sensors is explained as follows. As shown in Figs. 1D and 3F, the bristle structure resembles a cantilever beam structure. When a shear force *F*_13_ (i.e., the shear force is applied from channel 1 to channel 3) is applied at the tip of the bristles, the membrane in the region of channel 3 is compressed, resulting in better contact of the microcracks and the decrease in resistance of channel 3. On the contrary, the membrane in the region of channel 1 is stretched, causing the microcracks of channel 1 to separate from each other and the resistance of channel 1 to increase. Overall, the deflection of the bristle causes compression or tension of the polydimethylsiloxane (PDMS) membrane, and the microcracks on the membrane surface will contact or separate, respectively.

The change of microcrack channels caused by the shear force *F*_31_ is opposite to that caused by shear force *F*_13_, which give rise to opposite signal variations in these 2 states. Therefore, the direction of shear force can be determined by detecting and comparing the resistance signal outputs of the different microcrack channels. It should be noted that when the device is stimulated by a normal force, as shown in Figs. 1L and 3F, the force transferred to the root of the bristles will cause the downward depression of the membrane and the increase of the upper surface area of the membrane near the bristles. Thus, the gaps between microcracks near the root of the bristles will increase and the resistances increase as well.

Since the bristle structure can be regarded as a cantilever beam, it is known that the stress of the cantilever beam can be calculated by

$$\sigma =\frac{32\mathrm{FL}}{\pi d^{3}}$$ (1)

where *d*, *F*, and *L* are the diameter of bristle, the modulus of the mechanical stimuli, and the distance between the position of the applied stress and the bottom end of the cantilever beam. The strain can be obtained:

$$\varepsilon =\frac{\sigma }{E}$$ (2)

where *E* is the bristle’s Young's modulus. From Eqs. 1 and 2, the strain at the root of the bristle increases when increasing the force.

What is more, the simplified form of the normalized conductance of microcrack sensor can be written as the following equation [41]

$$S=\frac{1}{2}\left(1-\mathrm{erf}\left(\frac{\ln\left(\frac{\varepsilon }{\varepsilon_{0}}\right)}{\mu }\right)\right)$$ (3)

where *erf(x)* is the error function, *ε*_0_ and *μ* are fitting parameters, and *ε* is the strain. The above equations indicate that the conductivity of the microcrack channel is related to the strain, which depends on the intensity and direction of the force as well.

The proposed sensing mechanisms of the synergetic microcrack-bristle structure were simulated by finite-element analysis (FEA). As shown in Fig. 1G and H, bending the bristles by 2 shear forces in the opposite directions was simulated. The directions of the applied forces and the corresponding status of the microcrack-bristle structure are shown in Fig. S4. In the simulation results, prominent stress concentration can be observed in the microcracks around the bristle root part. Moreover, the unsymmetrical stress distribution can also be seen obviously. As shown in Fig. 1G, when a shear force from the right to left direction is applied, the microcracks on the left side are close to each other, and the microcracks on the right side are disconnected. Figure 1H shows the opposite situation of Fig. 1G. As shown in Fig. 1I, the strain distribution when the sensor is subjected to a normal force was also stimulated. The microcracks on both sides of the bristles separate from each other, and the channel conductivity decreases under a normal force, which shows completely different situation from the 2 cases mentioned above. These simulation results verify the feasibility of the microcrack-bristle structure design in distinguishing the directions of the externally applied force.

### Fabrication and characterization of the tactile sensors

**Fig. 2.**
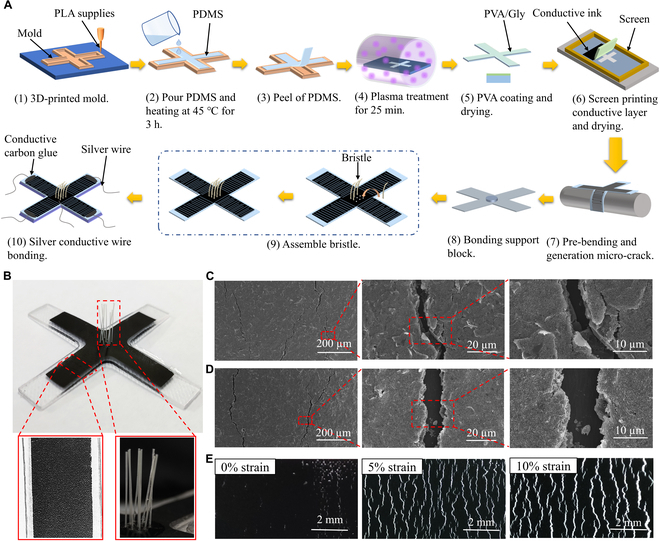
The fabrication process and morphology characterizations of the bioinspired tactile sensors based on the synergetic microcrack-bristle structure. (A) Schematic showing the fabrication process of the tactile sensors. (B) Photograph of the bioinspired tactile sensors based on the synergetic microcrack-bristle structure design. The inset is an enlarged view of the microcracked conductive channels and the bristle structures. (C and D) SEM images of the microcracked conductive channels at different magnifications without strain (C) and with 5% strain (D). (E) Optical images of the microcracked structure when subjected to different strains: 0% (left), 5% (middle), and 10% (right).

The fabrication process of the bioinspired tactile sensors based on the synergetic microcrack-bristle structure is illustrated in Fig. 2A. PDMS with good softness was selected as the material to prepare the membrane and the foundation of sensors. The cross-shaped membranes and foundations were obtained by pouring and curing PDMS into 3-dimensional (3D)-printed molds. In order to avoid delamination between the conductive carbon ink layer and the PDMS membrane, plasma treatment and polyvinyl alcohol/glycerin (PVA/Gly) film coating were used to improve the hydrophilicity of the PDMS membrane surface, thereby enhancing the adhesion of the conductive layer on the substrate. The conductive carbon ink pattern was prepared by screen printing on the plasma-treated membrane. Studies showed that the crack spacing (or density) can be regulated through pre-bending treatment with different curvature diameters [41]. As shown in Fig. S5, the strain sensitivity of the microcrack channels is affected by the crack density. Then, the microcrack sensing structures were obtained by pre-bending the partially dried brittle conductive carbon ink layer and the diameters of curvature of the pre-bending were set at 2 mm. As shown in Fig. 2A and Fig. S6, 9 nylon fibers, approximately 0.35 mm in diameter, were inserted into the center of the PDMS membrane. Finally, the PDMS foundation and PDMS membrane with microcrack structure were assembled with PDMS precursor followed by curing process. Another layer of conductive carbon ink was printed around the root of bristles as a shared reference electrode and printed at the end of each channel as measuring electrode. The preparation process proposed in this work has obvious advantages in terms of simple solution processing and superior cost-efficiency. Figure 2B presents a prepared bioinspired tactile sensor based on the synergetic microcrack-bristle structure. Through the cross-shaped device configuration design, combined with the high sensitivity of the microcrack structure and mechanical signal amplification of the bristle structure, the final bioinspired tactile sensors have unique ability to detect both the magnitude and the directions of mechanical forces.

Figure 2C and D and Fig. S7 show thescanning electron microscope (SEM) images of the microcracked conductive channels in the initial and tensile states, respectively. At 0% strain, although small gaps exist between the microcracks, the microcracks are not in full contact with each other, and there are still massive connecting points in the microcracks with abundant conductive pathways formed. When the microcrack channels are subjected to a tensile deformation, as shown in Fig. 2E and Fig. S8, the gaps between the microcracks increase gradually and the number of connecting points in the microcracks decreases quickly, resulting in remarkable decline in the channel conductivity.

### Performance evaluation of the tactile sensors

**Fig. 3.**
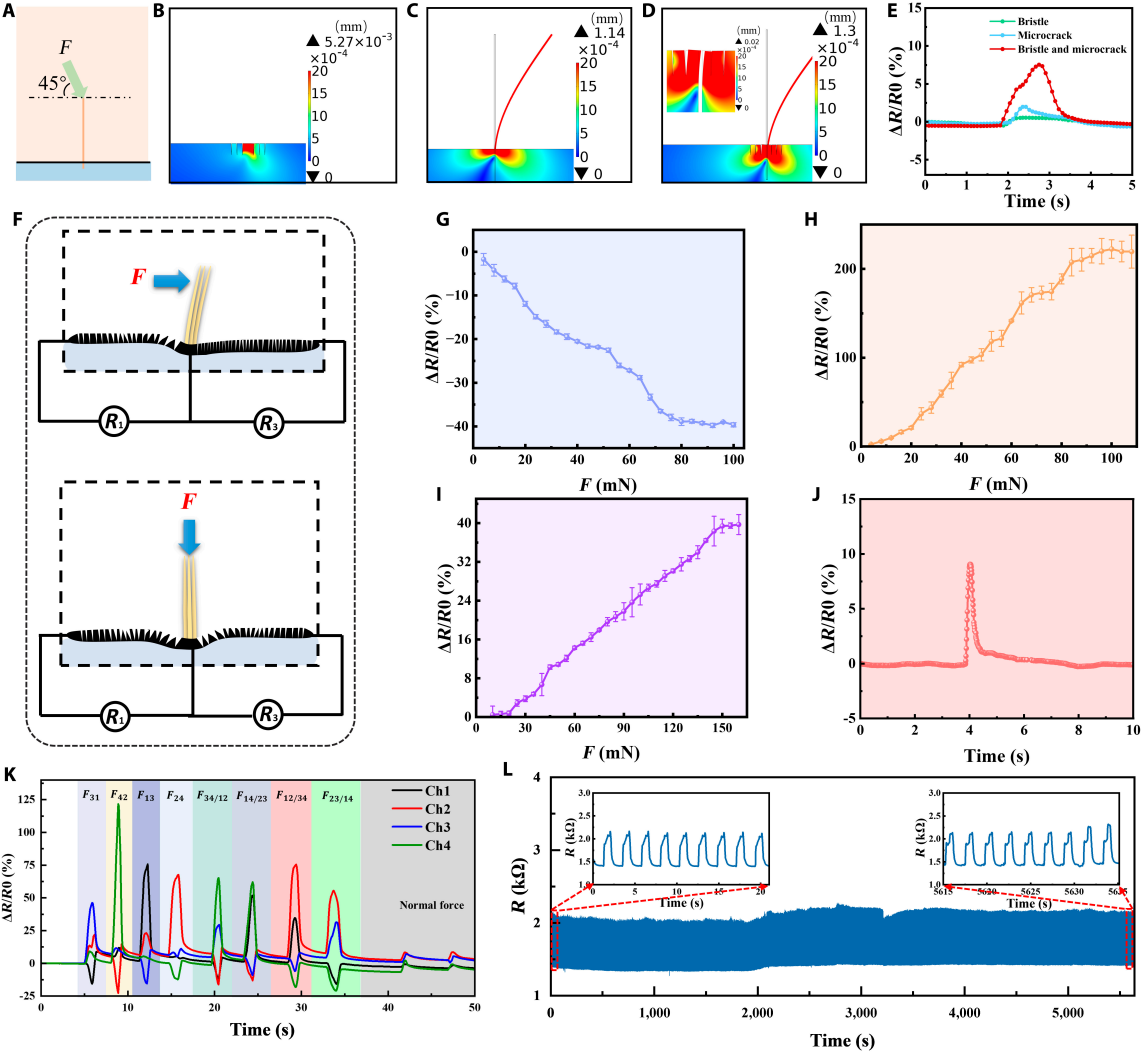
Sensing performance and response behaviors of the bioinspired tactile sensors based on synergetic microcrack-bristle structure. (A) Diagram showing a tilting force (*F*_x_ = 0.1 N, *F*_y_ = 0.1 N) of 45° direction applied on the device. (B to D) Finite element simulations illustrating the structure variations of the tactile sensors based on only microcrack structure (B), only bristle structure (C), and synergetic microcrack-bristle structure (D) with a 45° tilting force applied on the device. (E) Comparison of the response behaviors of the sensors based on microcrack structure, bristle structure, and synergetic microcrack-bristle structure under the 45° tilting force. (F) Schematics depicting the structure variations of the tactile sensor stimulated by a shear force and a normal force, respectively. (G to I) Response behaviors of the microcracked channel 3 (G) and microcracked channel 1 (H) under an increasing shear force *F*_13_ and under a normal force (I). (J) Response behavior of the tactile sensor when loading and unloading a tiny shear force of 5.4 mN. (K) Typical response behaviors of all of the 4 channels of the device when applying and removing shear forces of different directions and a normal force. (L) Stability test of the tactile sensors by loading–unloading a shear force (32 mN) for 2,500 cycles.

In order to verify the synergistic effect of the microcrack and bristle structure on the sensing performance of the tactile sensors, the stress distribution analysis and response behavior comparison of sensors with only microcrack structure, only bristle structure, and synergistic bristle-microcrack structure were conducted. As shown in Fig. 3A, a 45° tilting force (i.e., the direction of the force is 45° from the horizontal direction and 45° from the vertical direction, or *F_x_* = 0.1 *N*, *F_y_* = 0.1 *N*) was applied on the sensors, and the structure variations were simulated by finite element analysis. When stimulated by the tilting force, as presented in Fig. 3B to D, on the one hand, compared with sensor with only microcrack structure, sensor based on synergistic bristle-microcrack structure showed a wider strain distribution. On the other hand, compared with sensor with only bristle structure, the strain distribution in sensor based on synergistic bristle-microcrack structure was deeper. Therefore, the synergistic microcrack-bristle structure can significantly amplify the effect of mechanical stimulations and thus exhibit better mechanical susceptibility and sensitivity. In addition to finite element analysis, we further verified these results through rational experiments. As control samples, tactile sensors with only microcrack structure and only bristle structure were fabricated (see Materials and Methods for more details). When a tilting force of 12 mN is applied upon the devices with only microcrack structure and only bristle structure, as given in Fig. 3E, the magnitude of the response signal variations is much lower than that of the synergistic bristle-microcrack structure. This result clearly demonstrates the mutual enhancement effect of these 2 kinds of structure design. More specifically, the microcracks endow the tactile sensors with high sensitivity and the bristle structure further magnifies the mechanical sensitivity of the sensors.

In addition to the enhanced mechanical sensitivity, the combination of microcrack and bristle structures also endows the tactile sensors with distinctive capability of resolving the directions of the applied force. Figure 3F illustrates the different states of the microcrack structure and bristle structure when stimulated by a shear force or a normal force. When a shear force was loaded, the microcrack structure of the left sensing channel compressed toward each other, as shown in the left sensing channel of the upper Fig. 3F. The measured resistance value (*R*_3_) decreased when increasing the shear force *F*_13_ (Fig. 3G). On the contrary, the microcracks of the right sensing channel separated from each other as the substrate was stretched, as shown in the right sensing channel of the upper Fig. 3F. The resistance value (*R*_1_) increased when increasing the shear force *F*_13_ (Fig. 3H). When a normal force was loaded on the top of the tactile sensors, the bristles transmitted the mechanical stimulation to the root part. The elastic substrate around the bristle root would be convexly deformed, and the microcracks in the conductive channel would be separated as shown in Fig. 3F (bottom), resulting in an increase in the measured resistance, as confirmed in Fig. 3I.

Figure 3G to I and Fig. S9 show the relative resistance changes of channel 3 and channel 1 based on the synergistic microcrack-bristle structure under a shear force *F*_13_, *F*_14/23_ and under a normal force, respectively. The sensitivity of the tactile sensors can be defined as *S* = *δ*(*ΔR*/*R*_0_)/*δF*, where *ΔR*/*R*_0_ is the relative change in resistance and *F* is the applied force. Figure 3G shows the response curve of channel 3 when applying a shear force in the directions of 0°, and the resistance of the channel (*R*_3_ in Fig. 3F, top) decreases when increasing the 0° shear force magnitude (the sensitivity is −4.14 N^−1^ in the range of 0 to 80 mN). Figure 3H shows the response curve of channel 1 when subjected to a shear force in the directions of 180°. As the 180° shear force applied to the tactile sensor increases, the resistance of the channel (*R*_1_ in Fig. 3F, top) increases accordingly, and the sensitivity is 25.76 N^−1^ in the range of 0 to 100 mN. Moreover, it was also found that the sensitivity of the channel under the mechanical stimulations of 180° shear force was significantly higher than that under the 0° direction shear force. As shown in Fig. 3I, under the stimulation of a normal force, the resistance of the 4 sensor channels all increased gradually, and its sensitivity is 2.83 N^−1^ in the force range of 0 to 150 mN. These results demonstrate an ultrahigh sensitivity of the tactile sensor based on the synergy of microcracks and bristles in an appropriate shear force and normal force range. As shown in Fig. S10, we separately tested the response behaviors of microcrack channel 1 in 4 sensors when applying an increasing shear force. All the 4 sensors show similar response trends. However, the microcrack generation process and the consistency of the sensors could be improved further in future research.

**Fig. 4.**
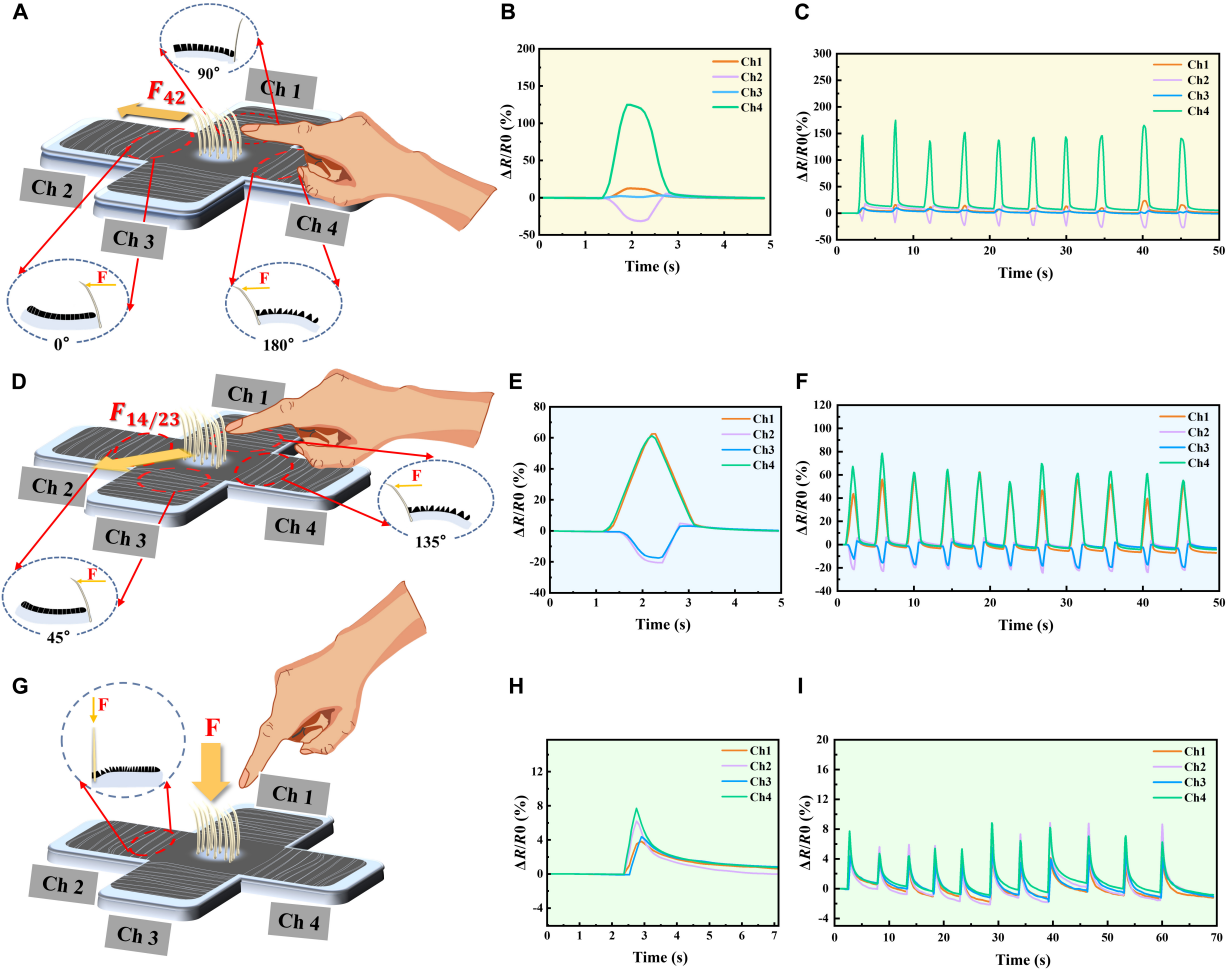
Resolving the directions of the applied force with the proposed tactile sensors. (A, D, and G) Schematic illustrations of the sensors subjected to shear forces *F*_42_ (A) and *F*_14/23_ (D) and normal force (G). (B, E, and H) Typical response behaviors of the 4 channels under shear forces of different directions: *F*_42_ (B), *F*_14/23_ (E), and normal force (H). (C, F, and I) Reliability test of 10 loading–unloading cycles under shear force *F*_42_ (C), *F*_14/23_ (F), and normal force (I).

In addition to high sensitivity and force direction-resolving capability of the sensors, the lowest detection limit of the sensors was also explored. Figure 3J shows the response curve of the sensor under a tiny shear force of 5.4 mN (Fig. S11), verifying the ultralow detection limit (<5.4 mN) of the proposed tactile sensors based on the synergetic microcrack-bristle structure. In addition, the response speed of the sensors was also tested. The results are shown in Fig. S12. The tactile sensors have fast response time (112 ms) and recovery time (279 ms). Figure 3K and Fig. S13 systematically verify the desirable response behaviors of the 4 sensor channels under different shear forces of various directions or under a normal force. By monitoring and analyzing the trend and the magnitude of signal changes of the 4 sensor channels simultaneously, the directions of the force applied to the sensors could be well distinguished (as verified in Figs. 3K and 4). In addition, the stability of the tactile sensor based on the synergetic microcrack-bristle structure was investigated by repeatedly loading and unloading a shear force of 32 mN on the device. As shown in Fig. 3L, the response signal variation of the sensors during 2,500 cycles test is relatively stable, exhibiting desirable repeatability and stability of the tactile sensors. What is more, as shown in Fig. S14, the responses of the sensor under different shear forces and at different rates in 10 cycles are almost consistent, indicating that the prepared tactile sensor has good cycle stability.

In order to further investigate the capability of the sensors to resolve the directions of the applied shear forces, we applied mechanical stimulations with different directions upon the tip of bristles (as shown in Fig. 4A, D, and G) and used a multichannel signal recording circuit board to monitor the resistance changes of the 4 channels of the tactile sensors. When applying a shear force *F*_42_ (i.e., 0°) to the sensors, as shown in Fig. 4B, the resistance of channel 4 increased, the resistance of channel 2 decreased, and the resistance of channel 1 and channel 3 changed very slightly. In addition, when applying a shear force *F*_14/23_ (i.e., 45°) to the sensors, the resistances of channel 1 and channel 4 increased (Fig. 4E), while the resistances of channel 2 and channel 3 decreased. When applying a normal force to the sensors, as shown in Fig. 4H, the resistances of the 4 channels all showed increases in the recorded resistance signals. As shown in Fig. 3F, the normal force would be transferred from the bristle tip to the root, thus exerting a downward force on the membrane and causing its deformation. Because the upper surface area of the membrane increased after deformation, microcracks would disconnect, causing resistance to increase.

Furthermore, we also verified the similar capability of the sensors in resolving other different forces in other directions, and the results are presented in Fig. S15. Besides, as shown in Fig. 4C, F, and I and Fig. S16, the response curves of the tactile sensors to different mechanical force directions exhibited high reliability and reproducibility during repeatedly loading–unloading same forces on the sensor over the 10 cycles continuously. Also, the prepared tactile sensor is also sensitive to vibration. As shown in Fig. S17A, when objects of different weights fell on the desk from a height of 10 cm and cause the desk to vibrate, the tactile sensor showed different response amplitudes correspondingly (Fig. S17B), which proved the ability of sensors to detect mechanical vibration of objects.

### Texture detection based on the tactile sensors

**Fig. 5.**
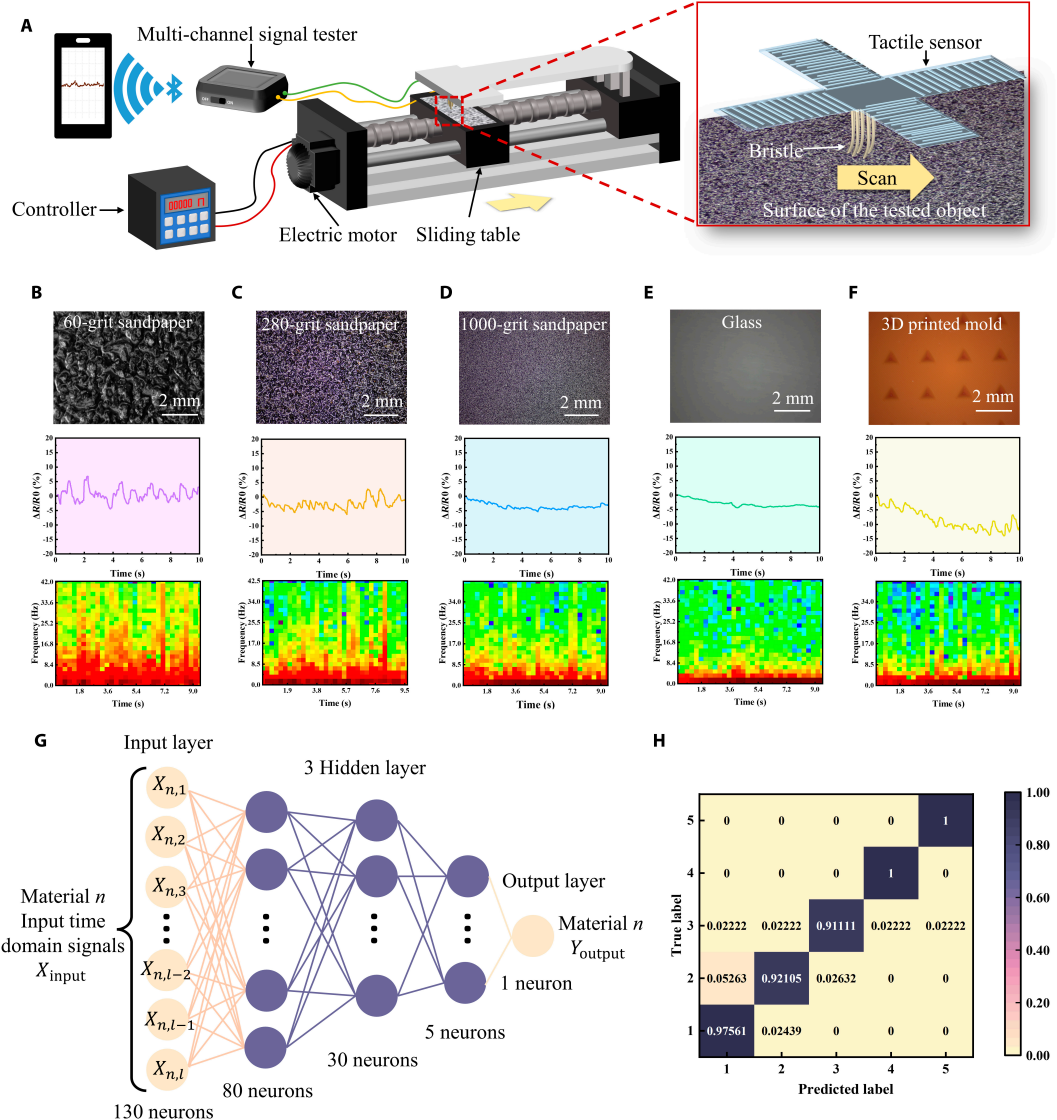
Texture detection based on tactile sensors with the synergetic microcrack-bristle structure. (A) Schematic illustration showing the measurement setup for the texture perception with the tactile sensors, in which the tactile sensor’s bristle structure scans along the surface of the objects with various textures. The left schematic showing the measurement equipment and the right illustration showing a magnified image of the bristle structure when it scans the surface of the objects. (B to F) Selected object surface with different texture situations. Optical images (top), relative resistance change signal outputs (middle), and STFT spectra (bottom) of (B) 60-grit sandpaper, (C) 280-grit sandpaper, (D) 1,000-grit sandpaper, (E) glass, and (F) 3D-printed mold with pyramid-shaped grooves (1 mm in length and depth). (G) Schematic diagram of the artificial neural network (ANN) used for texture recognition. The ANN consists of an input layer, 3 hidden layers, and an output layer where all the neurons between each layer are fully connected to each other. (H) Confusion matrix for texture predictions based on 200 groups of data in test dataset.

With high mechanical sensitivity, the proposed tactile sensors based on the synergetic microcrack-bristle structure can be used to detect and differentiate the surface texture of different objects. This is achieved by analyzing the temporal and spatial signals of the sensors when the sensors scan on the object surfaces. The measurement setup and working principle for texture recognition are shown in Fig. 5A. Objects with different surface roughness and textures were placed on a sliding table. An electric motor on the left side of the setup would drive the sliding table to move under a stable speed. The sliding speed could be well controlled by a programmed controller. As the sliding table moves from the left side to the right side at a stable speed of 2 mm/s, the bristles of the sensors will scan at a constant speed on the surface of objects. In this process, the surface texture of the tested objects would be reflected in the continuous response signals of the tactile sensors (e.g., frequency and intensity) by the highly sensitive synergetic microcrack-bristle structure. A multichannel signal tester was used to monitor the amplitude and period of the resistance changes of the sensors, and the texture of the objects can be detected and evaluated. It is worth pointing out that the scanned sensor signals are wirelessly transmitted to a smartphone app via Bluetooth, bringing more convenience for the practical application in surface texture detection.

Figure 5B to F shows the resistance signal variations of the sensors as it continuously scanned over sandpaper with different roughness, glass, and 3D-printed models with pyramid-shaped grooves, respectively. From the middle panels of Fig. 5B to F, it can be observed that when scanning rougher surfaces, such as 60-grit sandpaper, the resistance of the sensors changes with a larger magnitude (about 10% peak-to-peak). As the roughness of the sandpaper decreases, the amplitude variations of the output signal decrease correspondingly. When scanning a smooth surface (e.g., glass), the resistance response curve of the sensor was smooth as well.

In addition, STFT (short time Fourier transform) analysis of the resistance change signals was employed to further revolve the roughness of the object surfaces. As shown in the bottom row of Fig. 5B to F, different STFT characteristics can be extracted from the sensor response signals when the sensor was scanned on different textured surfaces. More specifically, rougher surfaces (e.g., 60-grit sandpaper) exhibited high amplitude features covering nearly all frequency range below 45 Hz. In comparison, the sensor signals obtained from smoother surfaces (e.g., glass) mainly cover the frequencies below 4 Hz. These results mentioned above reveal the great potential of the proposed tactile sensors based on the synergetic microcrack-bristle structures for texture detection and roughness differentiation application.

Besides, a machine learning framework is proposed to resolve and classify the signals obtained from the tactile sensor for recognition of different textures. We employed an artificial neural network (ANN) to construct the model, which consists of an input layer, 3 hidden layers, and an output layer (Fig. 5G). According to the classification results of 200 different groups of randomly selected samples, the confusion matrix is plotted as shown in Fig. 5H. The results reveal that the neural network model used for each recognition has a high accuracy (96.2%) for texture recognition, which verifies the good reliability of the machine learning framework and the bioinspired tactile sensor. All the above results show that the bioinspired tactile sensor can distinguish different surface textures, exhibiting promising application in the construction of electronic skin for smart robots.

### Biomimetic path explorations with the tactile sensor

**Fig. 6.**
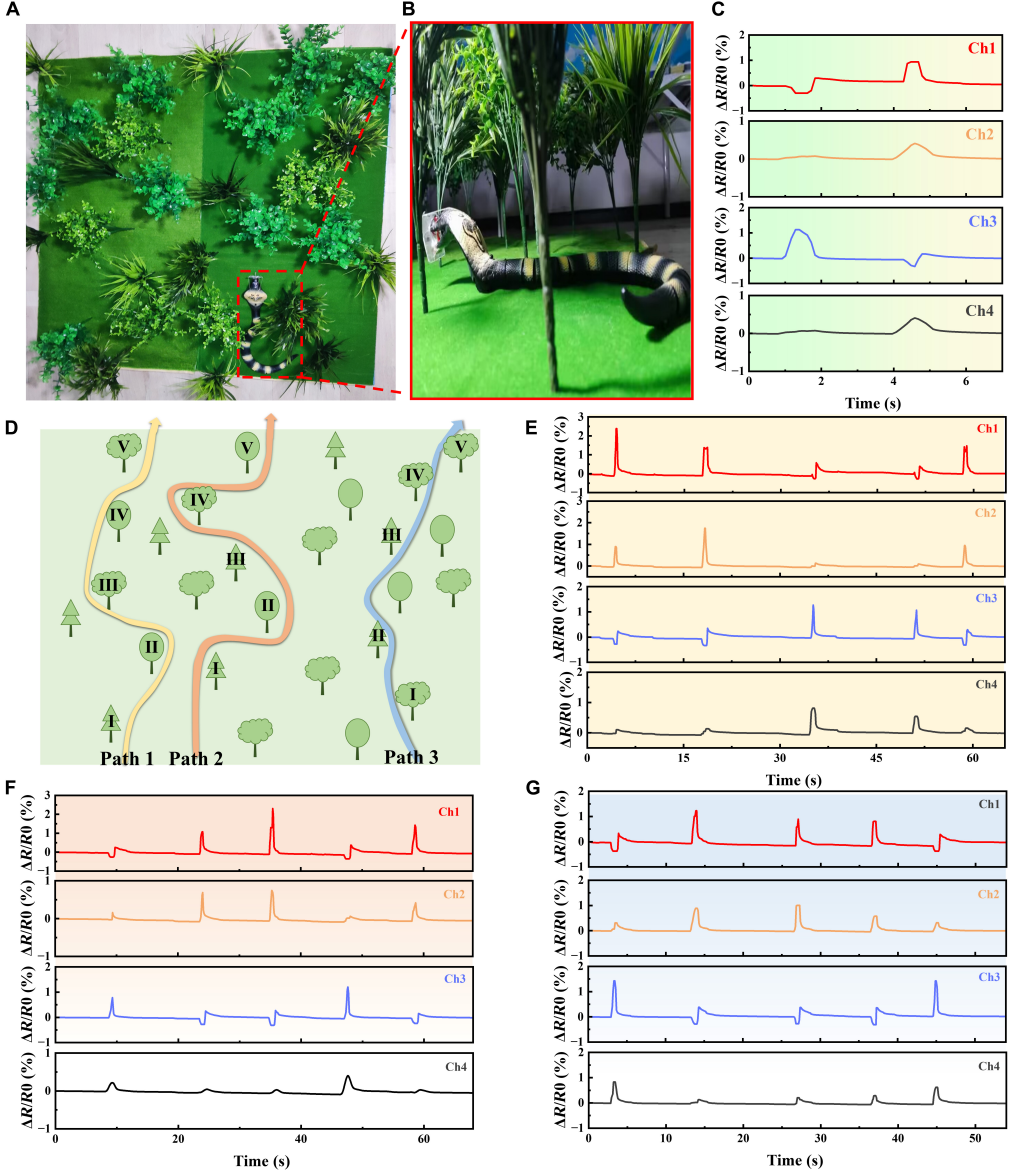
Path explorations with the developed tactile sensor based on synergistic microcrack-bristle structure. (A) Scene showing a model snake marching in the mimical jungle. (B) Picture showing the model snake equipped with a tactile sensor for path explorations. (C) Response behaviors of the sensor when the snake touches a plant on the left and right side, respectively. (D) Schematic diagram illustrating designed marching path in the mimical jungle. (E to G) Response behaviors of the tactile sensor when the snake passed through paths 1, 2, and 3, respectively.

In nature, most animals rely on vision to explore paths. But some nocturnal animals living in the dark tend to lose their sight (e.g., snails and snake). Therefore, other sensory organs are used to perceive the external environment, to make up for the shortcomings of their poor eyesight. For example, snails, one of the nocturnal animals, have very poor vision, only being able to see within 5 to 6 cm. They rely on tentacles to contact obstacles and explore their way forward. Therefore, they touch obstacles by antennae with tactile sensation cells to explore their ways. To exploit the application feasibility of the tactile sensors for biomimetic path explorations, a model nocturnal animal (e.g., a model snake was employed here) equipped with a tactile sensor was designed and the typical responsive signals were recorded when the snake marched along different paths in a jungle, as shown in Fig. 6A. As shown in Fig. 6B, the sensor was fixed at the front of the snake head. Channels 1 and 3 of the sensor were located on the left and right sides, respectively, and channel 2 and channel 4 were located on the upper and lower sides, respectively. As the model snake marched through the jungle, the sensor's bristle structure would touch the trunk of the plants from different directions, giving rise to different signal output combinations of the sensor channels.

Figure 6C shows the response behaviors of the 4 sensor channels as the snake passed by the left and right sides of a plant, respectively. When passing through the left side of the plant, the bristle structure was touched and bent to the left after contacting with the trunk. At this time, the microcrack at channel 1 on the left was reconnected, and its resistance decreased, while the resistance of channel 3 on the right increased. On the contrary, when passing through the right side of the plant, the microcrack at channel 3 reconnected and the resistance decreased, while the resistance of channel 1 increased.

As shown in Fig. 6D, 3 different paths were designed, and 5 plants (I to V) were distributed on each path. Figure 6E to G shows the response behaviors of the sensors when the snake marched through paths 1, 2, and 3, respectively. Taking path 1 as an example, the snake first passed through the right side of plants I and II, then passed through the left side of plants III and IV, then passed through the right side of plant V, and finally leaved the jungle. The recorded output signals of the sensor were shown in Fig. 6E. When passing through the right side of plants I and II, the resistance of channel 3 decreased and the resistance of channel 1 increased. When passing through plants III and IV from the left, the resistance of channel 1 decreased and the resistance of channel 3 increased. It can be noticed that, because the microcrack structure is highly sensitive, the response curve of resistance reduction appears to rebound when the bristle structure quickly detached from the trunk. Despite this signal rebounding, the typical response characteristics of the sensors can still be well distinguished when the snake passed through different paths. These results showed that tactile sensor based on synergistic microcrack-bristle structure can be used for biomimetic path discriminations and explorations for bionic applications.

## Discussion

In summary, we proposed and demonstrated a new category of bioinspired piezoresistive tactile sensors based on the synergetic microcrack-bristle structure design combined with cross-shaped configuration engineering. On the one hand, the high mechanical sensitivity of the microcrack structure and the mechanical amplification ability of the bristle structure significantly improve the sensitivity of the tactile sensor. On the other hand, the microcrack-bristle structure design combined with the cross-shaped device configuration engineering endows the tactile sensor with good capability to distinguish the direction of the applied mechanical forces. As demonstrations, the proposed tactile sensors can detect the surface roughness of different objects such as sandpaper, glass, and 3D-printed mold with pyramid-shaped grooves. Furthermore, the tactile sensor is fixed to a model snake to achieve effective discrimination and exploration of different paths. These results indicate great potential of the tactile sensors based on the synergistic microcrack-bristle structure for ingenious tactile sensing in various robotic and bionic application scenarios. The proposed tactile sensors are also expected to be applied to build human-like tactile system for robots and restore tactile perception in prosthetics.

## Materials and Methods

### Fabrication of the tactile sensors

First, the 3D printing molds (polylactic acid, Zhejiang Flashforge 3D Technology Co. Ltd., China) for casting membrane and foundation of sensors were designed by SolidWorks. The designed 3D models were imported into simplified 3D to generate G codes to determine the printing paths. The length, width, and depth of each channel of the cross-shaped mold 1 used to prepare the membrane are 1 mm, 40 mm, and 8 mm. Different from mold 1, the groove depth of mold 2 used to prepare the foundation is 2 mm, and there is a cross-shaped protrusion in the center (thickness is 1.3 mm, width is 4 mm, size is 20 × 20 mm). It is worth mentioning that the molds were printed at room temperature after transmitting the G code to an FDM 3D printer (Flashforge Dreamer NX, China).

Then, the PDMS precursor (Sylgard 184, Dow Corning Ltd.) was mixed with its curing agent at the ratio of 10:1 and then stirred for 5 min. The mixture was left for 20 min at room temperature to remove air bubbles. The PDMS was poured into the 3D-printed molds and cured in an oven at 45 °C for 3 hours. Then, the PDMS membrane and foundation were peeled off from the molds by tweezers.

The adhesion between PDMS and conductive carbon ink is weak, which is due to the high hydrophobicity and low surface energy of PDMS. Therefore, air was introduced into the plasma processor (VP-R, Guangzhou Sunjune Technology Co. Ltd., China) to make the surface hydrophilic for 25 min. Subsequently, an aqueous solution consisting of 2% (wt%) PVA (purchased from Chengdu Kelong Chemical Co. Ltd.) and Gly (provided by Chengdu Kelong Chemical Co. Ltd.) (the weight ratio of Gly to PVA was 8%) is coated onto PDMS membrane and dried at 60 °C for 20 min to further improve hydrophilicity. Then, a conductive carbon ink layer [CH-8, JUJO Printing Supplies & Technology (Pinghu) Co. Ltd., China] was screen printed on the PDMS membrane. To generate cracks uniformly on the brittle and conductive carbon ink layer, the 4 channels of the sensors were bent on a rod with a diameter of 2 mm to induce crack growth.

A support block (cured from PDMS, the size is 5 × 5 × 0.5 mm) was bonded to the center of the back of the PDMS membrane with liquid PDMS and placed it in the oven at 45 °C for 1 h. After the support block and the top PDMS substrate were bonded, a needle was used to poke 9 small holes in the middle of the membrane and 9 nylon fibers (the length and diameter are 10 mm and 0.35 mm, respectively) were inserted vertically into these holes as bristles (Fig. S6).

Next, the membrane and foundation were bonded using uncured PDMS. Then, a small amount of conductive carbon ink was coated around the root of bristles as reference electrodes and the tail parts of each channel as measuring electrodes, respectively. Note that the reference electrode was in the center of the cross-shaped conductive layer. Last, the silver wires were used as conductive wires and were bonded to each electrode through conductive carbon ink.

Compared with the tactile sensors based on the synergetic microcrack-bristle structure, the fabrication process of a device with microcrack structure but no bristle structure simply lacks the process of assembling the bristles. However, the conductive layer of sensors with only bristle structure was prepared using different fabrication processes and materials. Carbon nanotubes (CNTs) were chosen as the conductive layer material. Two grams of CNT powder (Shenzhen Hongdachang Technology Co. Ltd., China) was dispersed in 20 g of 98% alcohol. The dispersions were sonicated for 30 min to mix well. Then, the dispersions were sprayed on the PDMS membrane. After the CNT conductive layer was dried for 30 min in an oven, a layer of PDMS was covered to prevent the conductive layer from falling off and generating unnecessary microcracks during the experiment. In addition to the preparation and materials of the conductive layer, sensors with only bristle structure have the same fabrication process as the tactile sensor based on the synergetic microcrack-bristle structure.

### Simulation of sensors

In order to investigate the working principle of the proposed synergy between microcrack and bristle structure, simulations were carried out using the finite element analysis (FEA) method. The finite element model was simplified to one bristle and 6 microcrack structures with the parameters of the microcrack surface width = 2 μm, bottom width = 0.1 μm, depth = 1 mm, Young's modulus *E* of PDMS ≈ 0.3 MPa, Poisson ratio ≈ 0.3, bristle length above the PDMS substrate *L* ≈ 8 mm, and hair diameter *d* ≈ 0.35 mm.

### Machine learning for texture detection

In order to evaluate the ability of sensors to detect the texture of different materials, a multilayer neural network is trained and classified based on the obtained original signals. The first 130 sampling points of each group of original signals are used as datasets for sample division for training and testing. For 5 different surface textures, a total of 5 × 100 groups of sample data were collected. The labels of the corresponding 5 types of data are identified by 0 to 4 and are one-hot encoded. Sixty percent of the samples and labels are taken as the training set and verification set. Forty percent of the samples and labels are taken as test set. The neural network structure is composed of 4 layers, with the number of neurons in each layer being 130, 80, 30, and 5, respectively (Fig. 5G). The output activation function of layers 1 to 3 is the ReLU function, and that of the last layer is the Softmax function. The loss function used in the training process is the cross-entropy function. We adopt the Adam optimizer for parameter optimization with an initial learning rate lr = 0.01. The maximum number of training rounds is set to 100, and the final model was obtained by 10-fold cross-validation method for classification testing. According to the classification results of 200 different groups of randomly selected samples, the confusion matrix is plotted as shown in Fig. 5H.

### Device characterization

The microscopic observations were performed on an optical microscope (SN-300, China). The SEM observations were characterized using Apero S HiVac (FEI, America). The resistance signals of the 4 channels of the sensors were simultaneously and wirelessly transmitted to the smartphone by using a portable precision multichannel resistance tester (LinkZill 01RC, China). The sensitivity and machinal stability tests were carried out on a custom-made setup with 2 sliding stages, a stepper motor, and a programmable motor controller (KH-01, China). The tactile sensor was fixed on a sliding stage, and the digital force gauge (HANDPI, HP-2) was fixed on the other sliding stage to ensure that the force measuring rod of the digital force gauge was vertically aligned with the bristle’s tip of the sensor. Then, the sliding table with sensors was controlled to move back and forth at a constant speed by inputting a program on the programmable motor controller. The external force was provided and displayed by the digital force gauge. With the increase of displacement, the applied mechanical force increases accordingly.

## Data Availability

All data needed to evaluate the conclusions in the paper are present in the paper and/or the Supplementary Materials.

## Supplementary Materials

Supplementary 1 Notes S1 to S3 Table S1 Figs. S1 to S17 References [44–58]

## References

[1] An BW, Heo S, Ji S, Bien F, Park J-U. Transparent and flexible fingerprint sensor array with multiplexed detection of tactile pressure and skin temperature. Nat Commun. 2018;9(1):Article 2458. 29970893 10.1038/s41467-018-04906-1 PMC6030134

[2] Shin J, Jeong B, Kim J, Nam VB, Yoon Y, Jung J, Hong S, Lee H, Eom H, Yeo J, et al. Wearable temperature sensors: Sensitive wearable temperature sensor with seamless monolithic integration. Adv Mater. 2020;32(2):Article 2070014. 10.1002/adma.201905527 31696977

[3] Liu H, Sun K, Guo X-L, Liu Z-L, Wang Y-H, Yang Y, Yu D, Li Y-T, Ren T-L. An ultrahigh linear sensitive temperature sensor based on PANI:Graphene and PDMS hybrid with negative temperature compensation. ACS Nano. 2022;16(12):21527–21535. 36449370 10.1021/acsnano.2c10342

[4] Ma L, Wu R, Patil A, Zhu S, Meng Z, Meng H, Hou C, Zhang Y, Liu Q, Yu R, et al. Full-textile wireless flexible humidity sensor for human physiological monitoring. Adv Funct Mater. 2019;29(43):Article 1904549.

[5] Lu Y, Yang G, Shen Y, Yang H, Xu K. Multifunctional flexible humidity sensor systems towards noncontact wearable electronics. Nanomicro Lett. 2022;14(1):Article 150. 35869398 10.1007/s40820-022-00895-5 PMC9307709

[6] Cao Y, Li T, Gu Y, Luo H, Wang S, Zhang T. Fingerprint-inspired flexible tactile sensor for accurately discerning surface texture. Small. 2018;14(16):Article e1703902. 29504238 10.1002/smll.201703902

[7] Li X, Fan YJ, Li HY, Cao JW, Xiao YC, Wang Y, Liang F, Wang HL, Jiang Y, Wang ZL, et al. Ultracomfortable hierarchical nanonetwork for highly sensitive pressure sensor. ACS Nano. 2020;14(8):9605–9612. 32692150 10.1021/acsnano.9b10230

[8] Wang H, Liang C, Zhang H, Diao Y, Luo H, Han Y, Wu X. Digitized construction of iontronic pressure sensor with self-defined configuration and widely regulated performance. Sensors (Basel). 2022;22(16):Article 6136. 36015893 10.3390/s22166136 PMC9415562

[9] Boutry CM, Negre M, Jorda M, Vardoulis O, Chortos A, Khatib O, Bao Z. A hierarchically patterned, bioinspired e-skin able to detect the direction of applied pressure for robotics. Sci Robot. 2018;3(24):Article eaau6924. 10.1126/scirobotics.aau6914 33141713

[10] Guo H, Tan YJ, Chen G, Wang Z, Susanto GJ, See HH, Yang Z, Lim ZW, Yang L, Tee BCK. Artificially innervated self-healing foams as synthetic piezo-impedance sensor skins. Nat Commun. 2020;11(1):Article 5747. 33184285 10.1038/s41467-020-19531-0 PMC7665015

[11] Chen J, Zhu Y, Chang X, Pan D, Song G, Guo Z, Naik N. Recent progress in essential functions of soft electronic skin. Adv Funct Mater. 2021;31(42):Article 2104686.

[12] Liu Q, Zhang Y, Sun X, Liang C, Han Y, Wu X, Wang Z. All textile-based robust pressure sensors for smart garments. Chem Eng J. 2023;454:Article 14032.

[13] Liang C, Jiao C, Gou H, Luo H, Diao Y, Han Y, Gan F, Zhang D, Wu X. Facile construction of electrochemical and self-powered wearable pressure sensors based on metallic corrosion effects. Nano Energy. 2022;104:Article 107954.

[14] Zhao Z, Tang J, Yuan J, Li Y, Dai Y, Yao J, Zhang Q, Ding S, Li T, Zhang R, et al. Large-scale integrated flexible tactile sensor Array for sensitive smart robotic touch. ACS Nano. 2022;16(10):16784–16795. 36166598 10.1021/acsnano.2c06432

[15] Sun Z, Zhu M, Shan X, Lee C. Augmented tactile-perception and haptic-feedback rings as human-machine interfaces aiming for immersive interactions. Nat Commun. 2022;13(1):Article 5224. 36064838 10.1038/s41467-022-32745-8 PMC9445040

[16] Bai N, Wang L, Wang Q, Deng J, Wang Y, Lu P, Huang J, Li G, Zhang Y, Yang J, et al. Graded intrafillable architecture-based iontronic pressure sensor with ultra-broad-range high sensitivity. Nat Commun. 2020;11(1):Article 209. 31924813 10.1038/s41467-019-14054-9 PMC6954251

[17] Shi Z, Meng L, Shi X, Li H, Zhang J, Sun Q, Liu X, Chen J, Liu S. Morphological engineering of sensing materials for flexible pressure sensors and artificial intelligence applications. Nanomicro Lett. 2022;14(1):141. 35789444 10.1007/s40820-022-00874-w PMC9256895

[18] Zhao S, Ahn J-H. Rational design of high-performance wearable tactile sensors utilizing bioinspired structures/functions, natural biopolymers, and biomimetic strategies. Mater Sci Eng: R: Report. 2022;148:Article 100672.

[19] Tao J, Bao R, Wang X, Peng Y, Li J, Fu S, Pan C, Wang ZL. Self-powered tactile sensor Array systems based on the triboelectric effect. Adv Funct Mater. 2018;29(41):Article 1806379.

[20] Wu X, Ahmed M, Khan Y, Payne ME, Zhu J, Lu C, Evans JW, Arias AC. A potentiometric mechanotransduction mechanism for novel electronic skins. Sci Adv. 2020;6(30):Article eaba1062. 32832659 10.1126/sciadv.aba1062 PMC7439546

[21] Wu X, Zhu J, Evans JW, Arias AC. A single-mode, self-adapting, and self-powered mechanoreceptor based on a potentiometric–triboelectric hybridized sensing mechanism for resolving complex stimuli. Adv Mater. 2020;32(50):Article 2005970. 10.1002/adma.202005970 33179325

[22] Huang Y, Fan X, Chen S-C, Zhao N. Emerging technologies of flexible pressure sensors: Materials, modeling, devices, and manufacturing. Adv Funct Mater. 2019;29(12):Article 1808509.

[23] Choong C-L, Shim M-B, Lee B-S, Jeon S, Ko D-S, Kang T-H, Bae J, Lee SH, Byun KE, Im J, et al. Highly stretchable resistive pressure sensors using a conductive elastomeric composite on a micropyramid array. Adv Mater. 2014;26(21):3451–3458. 24536023 10.1002/adma.201305182

[24] Ruth SRA, Beker L, Tran H, Feig VR, Matsuhisa N, Bao Z. Rational design of capacitive pressure sensors based on pyramidal microstructures for specialized monitoring of biosignals. Adv Funct Mater. 2019;30(29):Article 1903100.

[25] He F, You X, Wang W, Bai T, Xue G, Ye M. Recent progress in flexible microstructural pressure sensors toward human–machine interaction and healthcare applications. Small Methods. 2021;5(3):Article 2001041. 10.1002/smtd.202001041 34927827

[26] Yang JC, Kim J-O, Oh J, Kwon SY, Sim JY, Kim DW, Choi HB, Park S. Microstructured porous pyramid-based ultrahigh sensitive pressure sensor insensitive to strain and temperature. ACS Appl Mater Interfaces. 2019;11(21):19472–19480. 31056895 10.1021/acsami.9b03261

[27] Zhao T, Li T, Chen L, Yuan L, Li X, Zhang J. Highly sensitive flexible piezoresistive pressure sensor developed using biomimetically textured porous materials. ACS Appl Mater Interfaces. 2019;11(32):29466–29473. 31291082 10.1021/acsami.9b09265

[28] Jinfeng Y, Yanan M, Gang J, Sairao Z, Yang Y, Feng C, Zhang C, Minglei C, Yongchen X, Peizhi S, et al. Bionic MXene based hybrid film design for an ultrasensitive piezoresistive pressure sensor. Chem Eng J. 2021;431:Article 133458.

[29] Zhao X-F, Hang C-Z, Lu H-L, Xu K, Zhang H, Yang F, Ma R-G, Wang J-C, Zhang DW. A skin-like sensor for intelligent braille recognition. Nano Energy. 2019;68:Article 104346.

[30] Kim C-C, Lee H-H, Oh KH, Sun J-Y. Highly stretchable, transparent ionic touch panel. Science. 2016;353(6300):682–687. 27516597 10.1126/science.aaf8810

[31] Chun K-Y, Son YJ, Jeon E-S, Lee S, Han C-S. A self-powered sensor mimicking slow- and fast-adapting cutaneous mechanoreceptors. Adv Mater. 2018;30(12):Article e1706299. 29424032 10.1002/adma.201706299

[32] Lee H-K, Chung J, Chang S-I, Yoon E. Normal and shear force measurement using a flexible polymer tactile sensor with embedded multiple capacitors. J Microelectromech Syst. 2008;17:934–942.

[33] Zhang J, Yao H, Mo J, Chen S, Xie Y, Ma S, Chen R, Luo T, Ling W, Qin L, et al. Finger-inspired rigid-soft hybrid tactile sensor with superior sensitivity at high frequency. Nat Commun. 2022;13(1):Article 5076. 36038557 10.1038/s41467-022-32827-7 PMC9422944

[34] Du L, Zhu X, Zhe J. An inductive sensor for real-time measurement of plantar normal and shear forces distribution. IEEE Trans Biomed Eng. 2015;62(5):1316–1323. 25546856 10.1109/TBME.2014.2386136

[35] Toyama S, Tanaka Y, Shirogane S, Nakamura T, Umino T, Uehara R, Okamoto T, Igarashi H. Development of wearable sheet-type shear force sensor and measurement system that is insusceptible to temperature and pressure. Sensors. 2017;17(8):1752. 28758986 10.3390/s17081752 PMC5579869

[36] Gu J, Kwon D, Ahn J, Park I. Wearable strain sensors using light transmittance change of carbon nanotube-embedded elastomers with microcracks. ACS Appl Mater Interfaces. 2019;12:10908–10917. 10.1021/acsami.9b18069 31877014

[37] Duan L, D'Hooge DR, Cardon L. Recent progress on flexible and stretchable piezoresistive strain sensors: From design to application. Prog Mater Sci. 2020;114:Article 100617.

[38] Fratzl P, Barth FG. Biomaterial systems for mechanosensing and actuation. Nature. 2009;462:442–448. 19940914 10.1038/nature08603

[39] Liu Y-F, Huang P, Li YQ, Liu Q, Tao JK, Xiong DJ, Hu N, Yan C, Wang H, Fu SY. A biomimetic multifunctional electronic hair sensor. J Mater Chem A. 2019;7(4):1889–1896.

[40] Barth, FG. In: Lehre M, editor. *Orientation and communication in arthropods*. Basel: Birkhäuser; 1997. p. 247–272.

[41] Kang D, Pikhitsa PV, Choi YW, Lee C, Shin SS, Piao L, Park B, Suh KY, Kim TI, Choi M. Ultrasensitive mechanical crack-based sensor inspired by the spider sensory system. Nature. 2014;516:222–226. 25503234 10.1038/nature14002

[42] Gong S, Yap LW, Zhu B, Zhai Q, Liu Y, Lyu Q, Wang K, Yang M, Ling Y, Lai DTH, et al. Local crack-programmed gold nanowire electronic skin tattoos for in-plane multisensor integration. Adv Mater. 2019;31(41):1903789. 10.1002/adma.201903789 31448484

[43] Yin B, Liu X, Gao H, Fu T, Yao J. Bioinspired and bristled microparticles for ultrasensitive pressure and strain sensors. Nat Commun. 2018;9:5161. 30514869 10.1038/s41467-018-07672-2 PMC6279775

[44] Noda K, Hoshino K, Matsumoto K, Shimoyama I. A shear stress sensor for tactile sensing with the piezoresistive cantilever standing in elastic material. Sensors Actuators A Phys. 2006;127(2):295–301.

[45] Man J, Zhang J, Chen G, Xue N, Chen J. A tactile and airflow motion sensor based on flexible double-layer magnetic cilia. Microsyst Nanoeng. 2023;9:12. 36688091 10.1038/s41378-022-00478-9 PMC9845383

[46] Dvorak N, Chung K, Mueller K, Ku P-C. Ultrathin tactile sensors with directional sensitivity and a high spatial resolution. Nano Lett. 2021;21(19):8304–8310. 34597518 10.1021/acs.nanolett.1c02837

[47] Park J, Lee Y, Hong J, Lee Y, Ha M, Jung Y, Lim H, Kim SY, Ko H. Tactile-direction-sensitive and stretchable electronic skins based on human-skin-inspired interlocked microstructures. ACS Nano. 2014;8(12):12020–12029. 25389631 10.1021/nn505953t

[48] Li S, Chen X, Li X, Tian H, Wang C, Nie B, He J, Shao J. Bioinspired robot skin with mechanically gated electron channels for sliding tactile perception. Sci Adv. 2022;8(48):Article eade0720. 36459548 10.1126/sciadv.ade0720 PMC10936060

[49] Harada S, Kanao K, Yamamoto Y, Arie T, Akita S, Takei K. Fully printed flexible fingerprint-like three-axis tactile and slip force and temperature sensors for artificial skin. ACS Nano. 2014;8(12):12851–12857. 25437513 10.1021/nn506293y

[50] Viry L, Levi A, Totaro M, Mondini A, Mattoli V, Mazzolai B, Beccai L. Flexible three-axial force sensor for soft and highly sensitive artificial touch. Adv Mater. 2014;26(17):2659–2664. 24677245 10.1002/adma.201305064 PMC4264044

[51] Nie B, Geng J, Yao T, Miao Y, Zhang Y, Chen X, Liu J. Sensing arbitrary contact forces with a flexible porous dielectric elastomer. Mater Horiz. 2021;8(3):962–971. 34821327 10.1039/d0mh01359e

[52] Hu J, Qiu Y, Wang X, Jiang L, Lu X, Li M, Wang Z, Pang K, Tian Y, Zhang W, et al. Flexible six-dimensional force sensor inspired by the tenon-and-mortise structure of ancient Chinese architecture for orthodontics. Nano Energy. 2022;96:Article 107073.

[53] Zeng X, Liu Y, Liu F, Wang W, Liu X, Wei X, Hu Y. A bioinspired three-dimensional integrated e-skin for multiple mechanical stimuli recognition. Nano Energy. 2022;92:Article 106777.

[54] Chen S, Bai C, Zhang C, Geng D, Liu R, Xie Y, Zhou W. Flexible piezoresistive three-dimensional force sensor based on interlocked structures. Sensors Actuators A Phys. 2021;330:Article 112857.

[55] Yin J, Santos VJ, Posner JD. Bioinspired flexible microfluidic shear force sensor skin. Sensors Actuators A Phys. 2017;264:289–297.

[56] Zhang J, Hao L, Yang F, Jiao W, Liu W, Li Y, Wang R, He X. Biomimic hairy skin tactile sensor based on ferromagnetic microwires. ACS Appl Mater Interfaces. 2016;8(49):33848–33855. 27960407 10.1021/acsami.6b14236

[57] He T, Liang Q, Zhang K, Mu X, Luo T, Wang Y, Luo G.A modified microfluidic chip for fabrication of paclitaxel-loaded poly(l-lactic acid) microspheres. Microfluid Nanofluid. 2011;10:1289–1298.

[58] Trantidou T, Elani Y, Parsons E, Ces O. Hydrophilic surface modification of PDMS for droplet microfluidics using a simple, quick, and robust method via PVA deposition. Microsyst Nanoeng. 2017;3:Article 16091. 31057854 10.1038/micronano.2016.91 PMC6444978

