# Supplementary material for: Bioinspired Tactile Sensation Based on Synergistic Microcrack-Bristle Structure Design toward High Mechanical Sensitivity and Direction-Resolving Capability
Source: Research (Wash D C). 2023 Jun 16;6:0172. doi: 10.34133/research.0172 (PMC10275619; doi:10.34133/research.0172)
Supplement: Supplementary 1 — Notes S1 to S3 Table S1 Figs. S1 to S17 References [44–58] [file research.0172.f1.docx]

Supplementary Information for

**Bioinspired Tactile Sensation based on Synergistic Microcrack-Bristle Structure Design towards High Mechanical Sensitivity and Direction-Resolving Capability**

Yiqun Zhang^1, 2^, Qi Liu^1, 2^, Wenjuan Ren^1^, Yangyang Song^1, 2^, Hua Luo^1^, Yangyang Han^3^, Liang He^1^, Xiaodong Wu^1^*, and Zhuqing Wang^1, 2^*

^1^ School of Mechanical Engineering, Sichuan University, Chengdu 610065, China

^2^ Med+X Center for Manufacturing, West China Hospital, Sichuan University, Chengdu 610041, China

^3^State Key Laboratory of Polymer Materials Engineering, Polymer Research Institute of Sichuan University, Chengdu 610065, China

*Address correspondence to: xiaodong_wu@scu.edu.cn; [wzhuqing@scu.edu.cn](mailto:wzhuqing@scu.edu.cn)

**Table of Contents**

**SENSING MECHANISMS AND STRUCTURES3**

Supplementary Note S13

Table S1. Comparison of our tactile sensor with the existing bioinspired tactile sensors 4

Fig. S1. Comparison of our tactile sensor with existing bioinspired tactile sensors5

Supplementary Note S25

Fig. S2. The force is transferred to the microcracks when the bristles are stressed6

Fig. S3. Schematic diagram showing the typical signal outputs from the four channels when apply different mechanical forces 7

Fig. S4. Schematic diagrams showing the structure variations when a shear force is applied 8

**CHARACTERIZATIONS OF THE SENSOR9**

Fig. S5. The densities of microcracks under pre-treatment with different pre-bending diameters and their effect on the performance of the sensor 10

Fig. S6. Dimensions and distribution spacing of bristles assembled on the PDMS membrane.11

Fig. S7. SEM images of the microcracked conductive channels12

Fig. S8. The statistic spacing between the corresponding cracks under different tensile strains.13

Fig. S9. Response behaviors of the microcracks channels14

Fig. S10. Similar response behaviors of the four sensors under an increasing shear force 15

Fig. S11. A tiny shear force variation applied to tactile sensor 16

Fig. S12. Response time and recovery time of the tactile sensors 17

Fig. S13. Typical response behaviors of the four sensor channels 18

Fig. S14. Responses of the sensor under different shear forces (A) and at different rates (B) in ten cycles test19

Fig. S15. Typical response behaviors of the four sensor channels under shear forces 20

Fig. S16. Reliability test of the sensors 21

**FABRICATION METHOD AND ITS APPLICATIONS 22**

Fig. S17. Schematic diagram and response curve of the sensor for detecting different vibration intensity 22

Supplementary Note S3 23

**Supplementary Note S1:**

During the long evolution, spiders rely on their highly evolved sensory organs to adapt to environmental changes. The mechanical sensory organs of spiders are extremely sensitive to mechanical signals. Based on the vibration signals transmitted by the ground or spider web, they can distinguish the location of the prey and the natural enemies and take action accordingly. Study has shown that spiders have two main types of sensory organs used for mechanical perception: slit sensilla and hair-like structures.

Slit organs are very sensitive vibration detectors embedded in the spider's exoskeleton, which are capable of measuring deformations of cuticular caused by mechanical loads from internal sources (such as muscle activity or hemolymph pressure), or from external sources (such as substrate vibration).

There are massive innervated cuticular bristles at the surface of a spider, which are ultra-sensitive mechanical sensing organs. External forces (such as contact forces and air movement) deflect the hair shaft, which rotates around an axis near its base. The dendrites of the three sensory cells terminate near the inner end of the hair shaft. They can send electrical signals to the central nervous system about the characteristics of stimulation (especially the occurrence and speed of hair deflection) and realize the detection of external stimuli^38^.

As a result of these two sensory organs with high sensitivity, the spider is highly sensitive to changes in the surrounding environment and can respond quickly. Inspired by the slit organs and bristles of spiders, based on the highly sensitive property of microcrack structure and the amplification and transmission effect of bristle structure, a tactile sensor is proposed in this work to resolve not only the intensity but also the directions of the external mechanical stimulations. As shown in Figure 3E, the synergistic effect of the microcrack-bristle structure was well verified. The magnitude of the response signal variation of the sensor with microcrack and bristle structure is much higher than that of the sensor with only microcrack structure and only bristle structure. This result clearly proved the good mutually enhancement effect of these two kinds of structure design.

**Table S1. Comparison of tactile sensors for resolving both mechanical intensity and directional features**

| **Sensors** | **Structure design** | **Sensing principle** | **Fabrication method** | **Performance** | **Refs** |
| --- | --- | --- | --- | --- | --- |
| **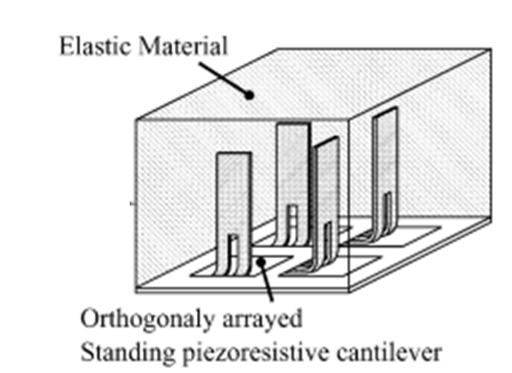**  **Sensor 1** | Piezoresistive cantilever beam array with orthogonal distribution. | Piezoresistive | - Photolithography - Rapid thermal diffusion - Deep reactive ion etcher (DRIE)) | - Detection limit: None - Sensitivity: 1.30 ×10^-3^ kPa^-1^ - Shear force detection: Yes - Normal force detection: No - Force direction detection: Yes | ^44^ |
| **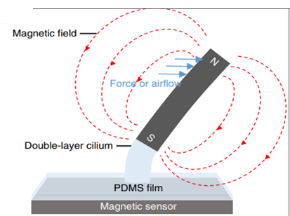**  **Sensor 2** | A double-layer magnetic cilia sensor. | Magnetic | - A metal tube pressing (MTP) method | - Detection limit: 2.1 μN - Sensitivity: 0.63 T/N - Shear force detection: Yes - Normal force detection: Yes - Force direction detection: No | ^45^ |
| **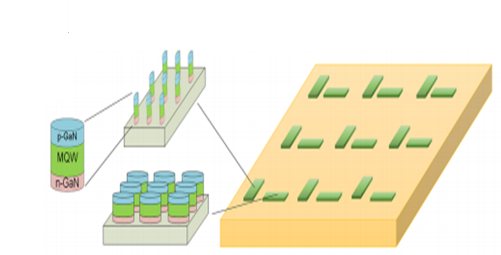**  **Sensor 3** | A sensor node was comprised of two orthogonal sensors made of GaN nanocolumns. | Optical | - Metal–organic chemical vapor deposition (MOCVD) - Electron beam lithography | - Detection limit: None - Sensitivity: None - Shear force detection: Yes - Normal force detection: No - Force direction detection: Yes | ^46^ |
| **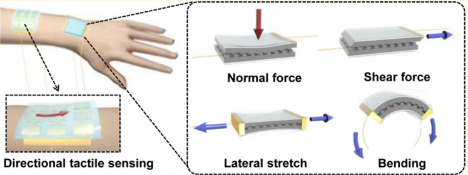**  **Sensor 4** | Bioinspired piezoresistive interlocked microdome array | Piezoresistive | - Solution casting - Sputter coating | - Detection limit: None - Sensitivity：2.21 N^-1^ - Shear force detection: Yes - Normal force detection: Yes - Force direction detection: No | ^47^ |
| **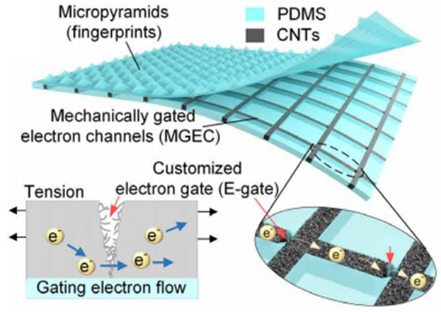**  **Sensor 5** | An electronic gate consisting of a mechanically gated electron channels (a custom V-shaped cracks within embedded mesh electron channels) and an external micropyramidal artificial fingerprint. | Piezoresistive | - Photolithography etching - Inductively coupled plasma chemical vapor deposition - Casting - Anisotropic potassium hydroxide etching - Spin coating - Oxygen plasma - Prestretching | - Detection limit: None - Sensitivity: None - Shear force detection: Yes - Normal force detection: Yes - Direction of the force detection: No | ^48^ |
| **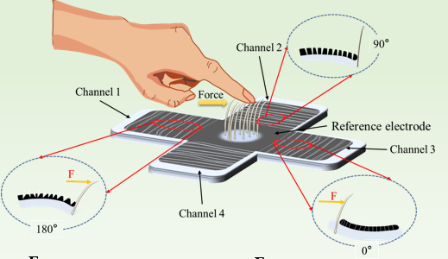**  **Sensor of this work** | Microcrack-bristle structure design combined with the cross-shaped device configuration engineering. | Piezoresistive | - Casting - Plasma - Screen coating - Prestretching | - Detection limit: 5.4 mN - Sensitivity: 25.76 N^-1^ - Shear force detection: Yes - Normal force detection: Yes - Force direction detection: Yes | This work |

**
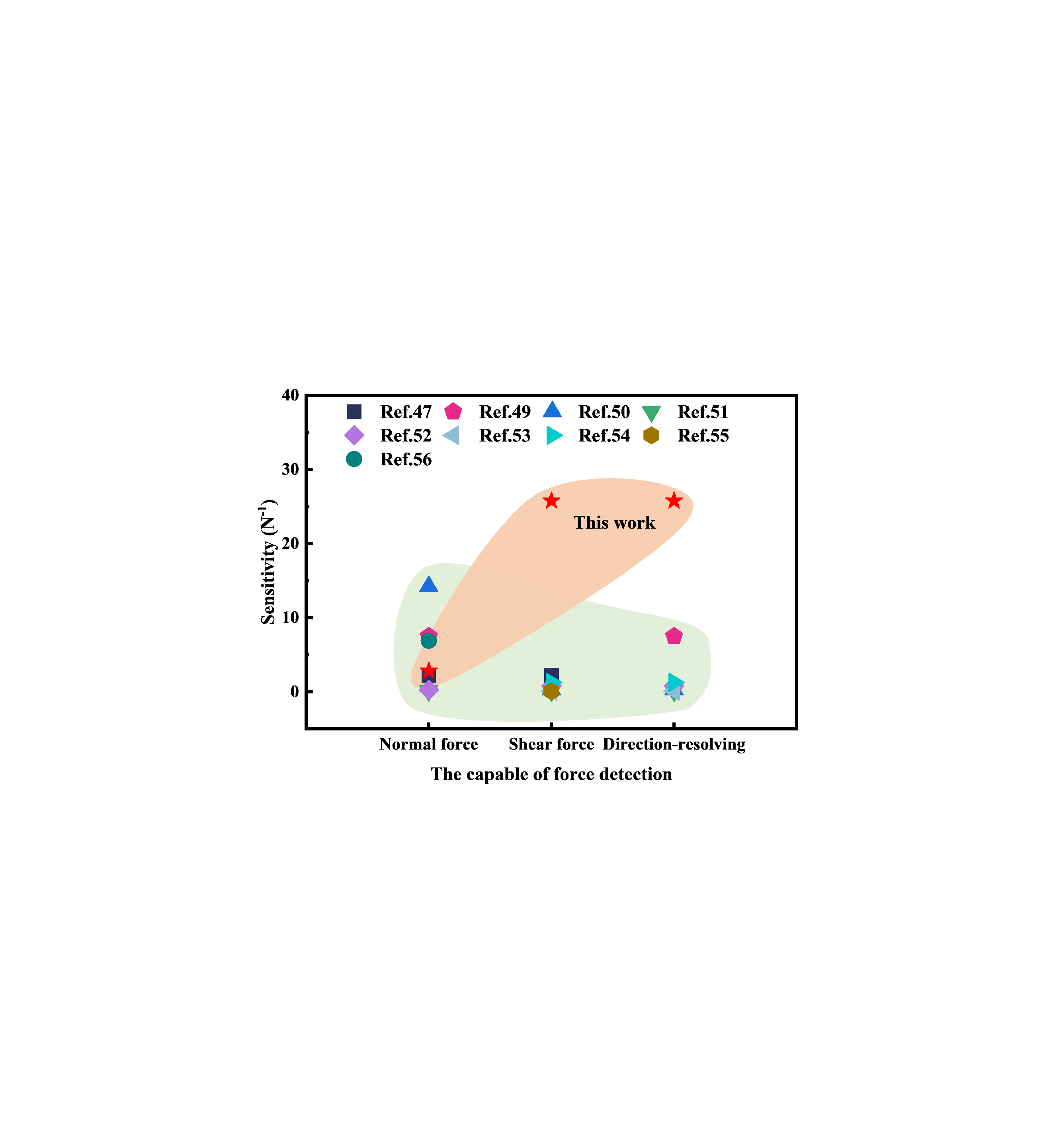
**

**Fig. S1. Comparison of our tactile sensor with the existing bioinspired tactile sensors**^47,49-56^**.**

**Supplementary Note S2:**

As shown in Fig. S2, we compared the direction-resolving capability and mechanical sensitivity of the sensor in this work with other reported bioinspired tactile sensors. The sensor based on the synergistic effect of microcrack-bristle structure can not only resolve the direction of shear force, but also has a high sensitivity for both shear force and normal force detection. In addition, Table S1 compares the design concept, working mechanism, and performance of our proposed sensor with other reported tactile sensors that are capable of detecting shear forces. All of the above sensors can detect shear force, but some sensors cannot detect normal force or the force direction, and can only obtain the modulus of shear force^44,46-48^. In addition, some haptic sensors necessitate commercially available Hall sensors or imaging optics to detect signals^45,46^. Others require sensor arrays to detect the force direction^47^. In this work, our tactile sensor based on the synergistic microcrack-bristle structure design and cross-shaped device configuration engineering can not only detect both shear force and normal force, but also can distinguish the direction of the shear forces. Primarily, it exhibits high mechanical sensitivity (25.76 N^-1^) and low detection limit (5.4 mN). What’s more, the preparation process proposed in this work has obvious advantages in terms of simple solution processing and superior cost-efficiency. The above sensors do not simultaneously have these performances. Hence, compared with various existing tactile sensors, the sensor based on synergistic microcrack-bristle structure exhibits a unique advantage in resolving both mechanical intensity and directional features. These results indicate that the proposed tactile sensor is expected to be applied in the construction of human-like tactile system, and has great application potential in robot and bionic application scenarios.

**
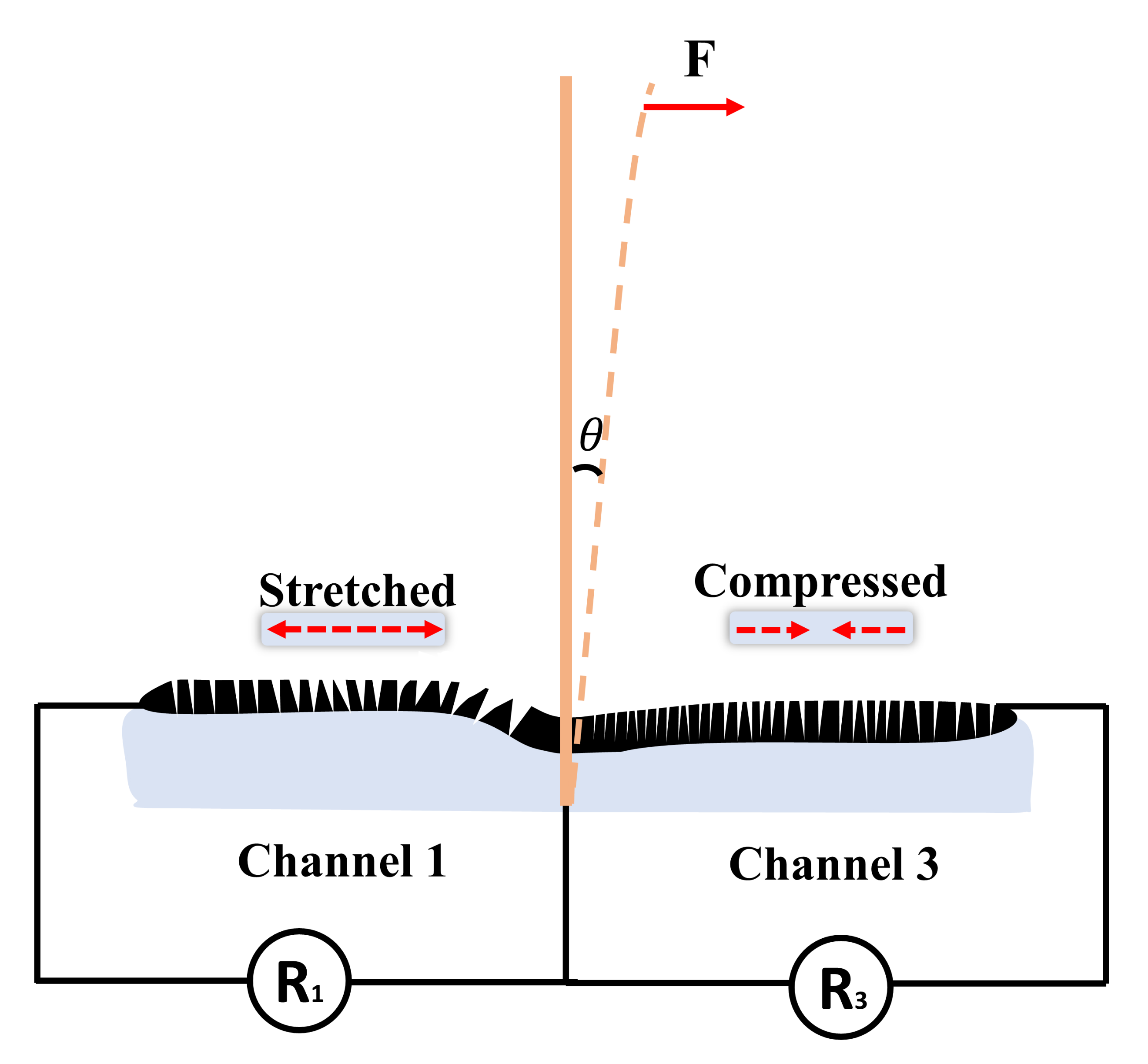
**

**Fig. S2. The force is transferred to the microcracks when the bristles are stressed.**

The bristle is similar to a cantilever beam, which acts as a mechanical signal transmission and amplification device. As shown in Fig. S2, when a shear force F is applied to the bristle tip, it causes the bristle to deflect by an angle $\theta$. The resulting force (F) is transferred to the root of bristle, causing strain on the PDMS membrane near the root. Then, the membrane of channel 3 is compressed, microcracks of channel 3 come in better contact and the resistance of channel 3 decreases. Conversely, the membrane in the region of channel 1 is stretched, causing the microcracks of channel 1 to separate from each other and the resistance of channel 1 to increase. Overall, the deflection of the bristle results in compression or tension of the PDMS membrane, and the microcracks on the membrane surface will contact or separate respectively. By detecting the signal response behavior of each microcrack channel simultaneously, the magnitude and direction of external mechanical stimuli can be obtained.


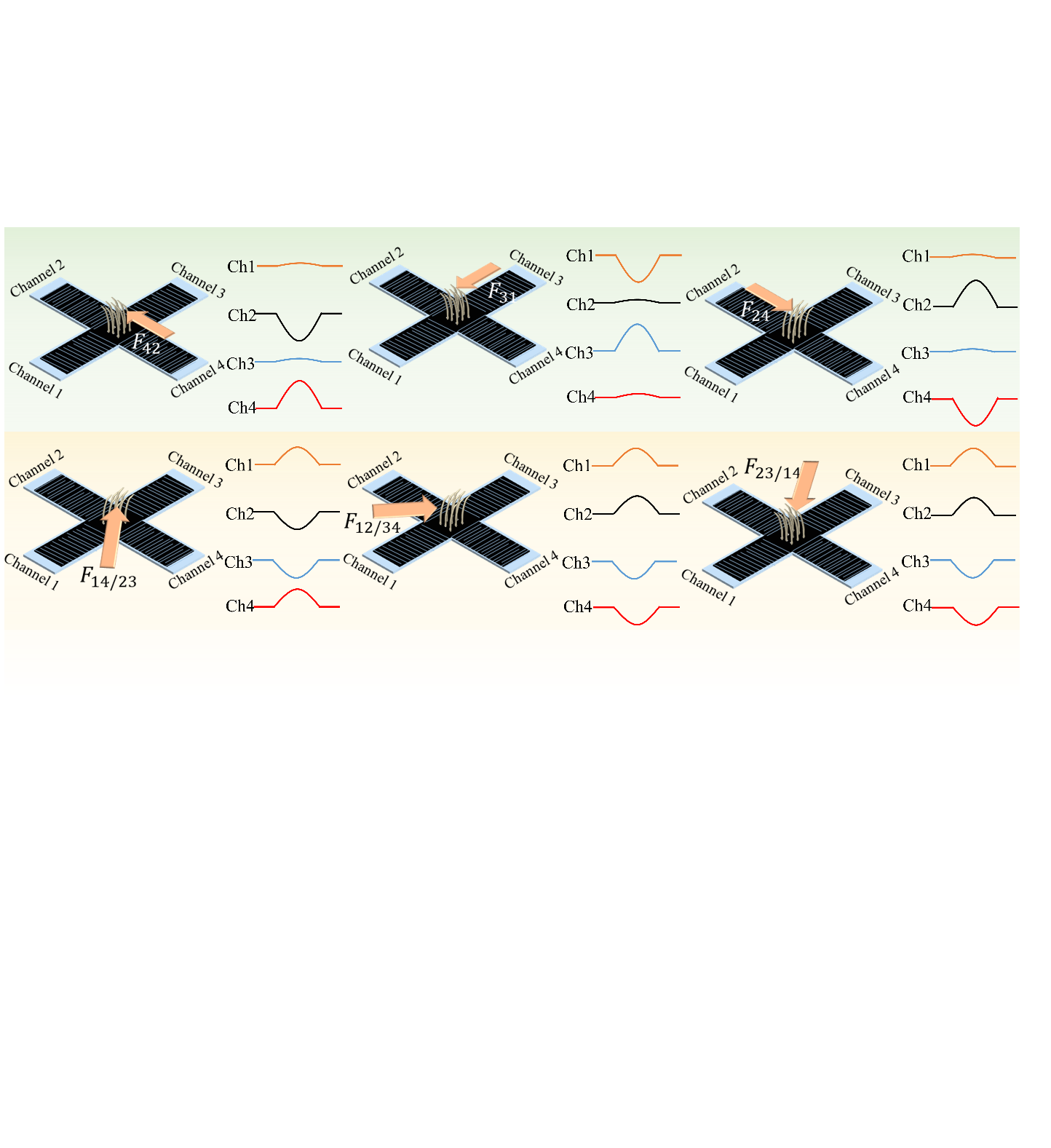


Fig. S3. Schematic diagram showing the typical signal outputs from the four channels when apply different mechanical forces.


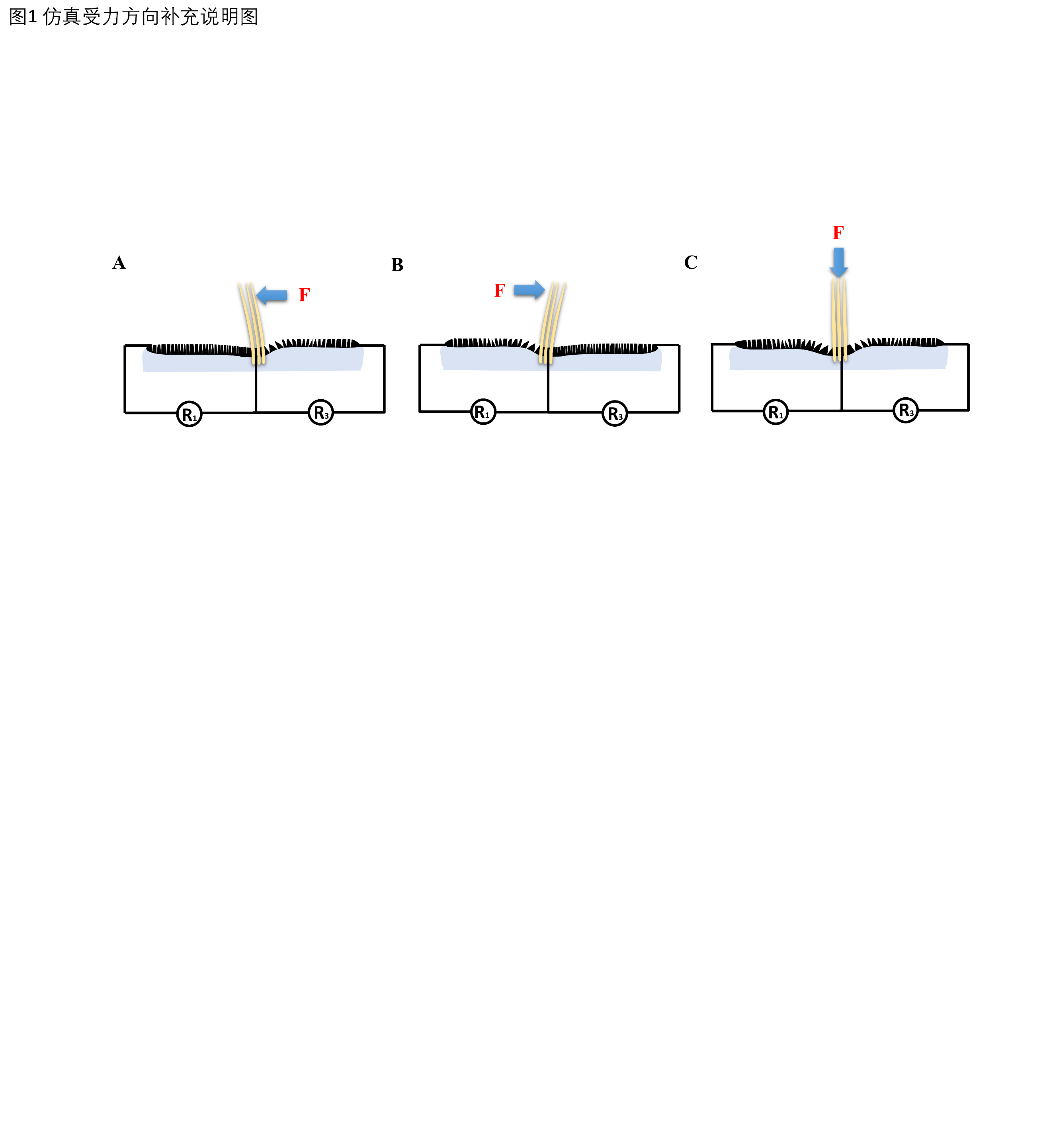


Fig. S4. Schematic diagrams showing the structure variations when a shear force is applied from right to left (A), from left to right (B), and a normal force (C) is applied from the top of the microcrack-bristle synergetic structure.


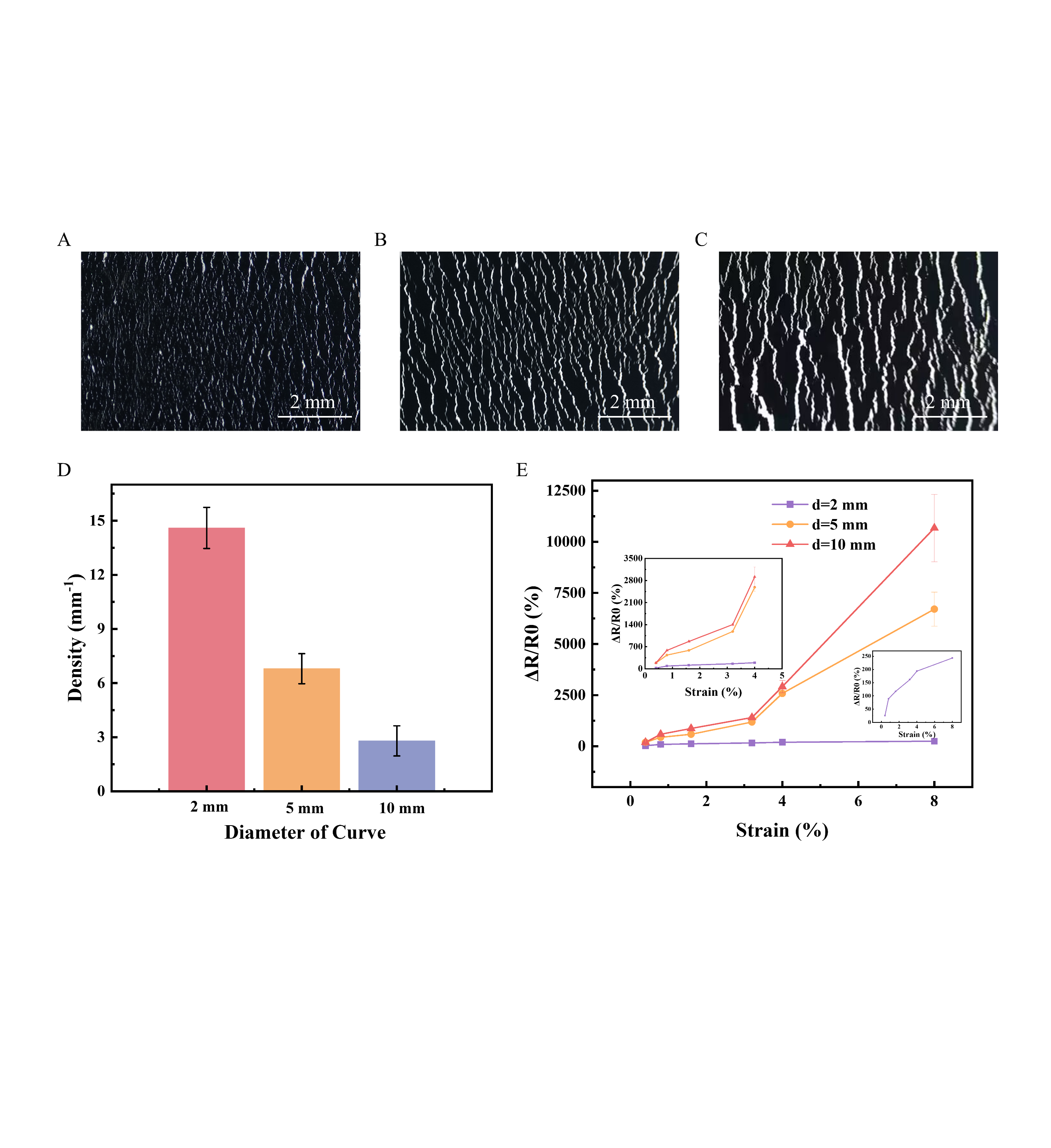


**Fig. S5. The densities of microcracks under pre-treatment with different pre-bending diameters and their effect on the performance of the sensor.** (A-C) Optical images of microcrack sensors under 3% strain by pre-bending with different diameters of 2 mm (A), 5 mm (B), and 10 mm (C). (D) Microcrack densities under different pre-bending diameters. (E) Sensitivity of sensors with different microcrack densities.

The number and size of the microcracks can be adjusted by changing the radius of curvature of the pre-bending^41^. As shown in Fig. S5A-D, the larger the radius of curvature, the larger the size and the fewer the number of microcracks. In addition, Fig. S5E compares the sensitivity of microcrack sensors with different densities. The results show that the smaller the curvature diameter, the larger the microcrack density, resulting in a smaller sensitivity and a larger sensing range.

Due to the inherent randomness of the microcrack structure, the microcracks in the four channels cannot be completely consistent. However, a pre-bending curvature diameter (d=2 mm) is strictly adopted in this work. Therefore, the microcrack density in the four channels is basically consistent, which can ensure that they exhibit approximate sensing performance.


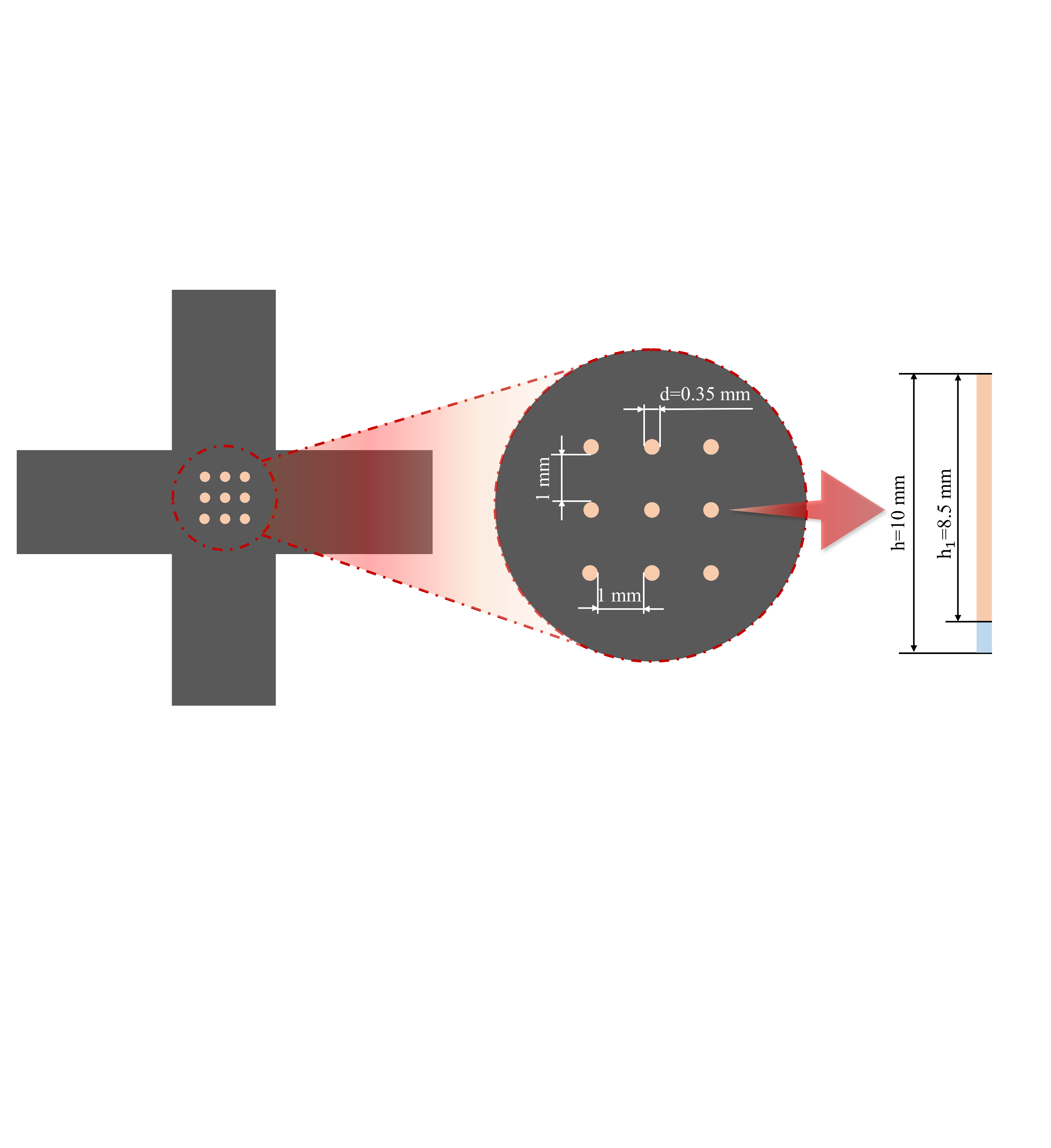


**Fig. S6.** **Dimensions and distribution spacing of bristles assembled on the PDMS membrane.**

The bristles consist of flexible nylon fibers that are assembled on the PDMS film. The whole assembling process of the bristles can be well quantized. As described in the experiment section, each bristle has a diameter of about 0.35 mm and a length of 10 mm. And the height of each bristle on the membrane is 8.5 mm. As shown in Fig. S6, vertical 3 x 3 bristles are assembled on the membrane in an array, with each bristle spaced 1 mm apart. Therefore, the whole preparation process can be quantized and controlled to reduce the variation.


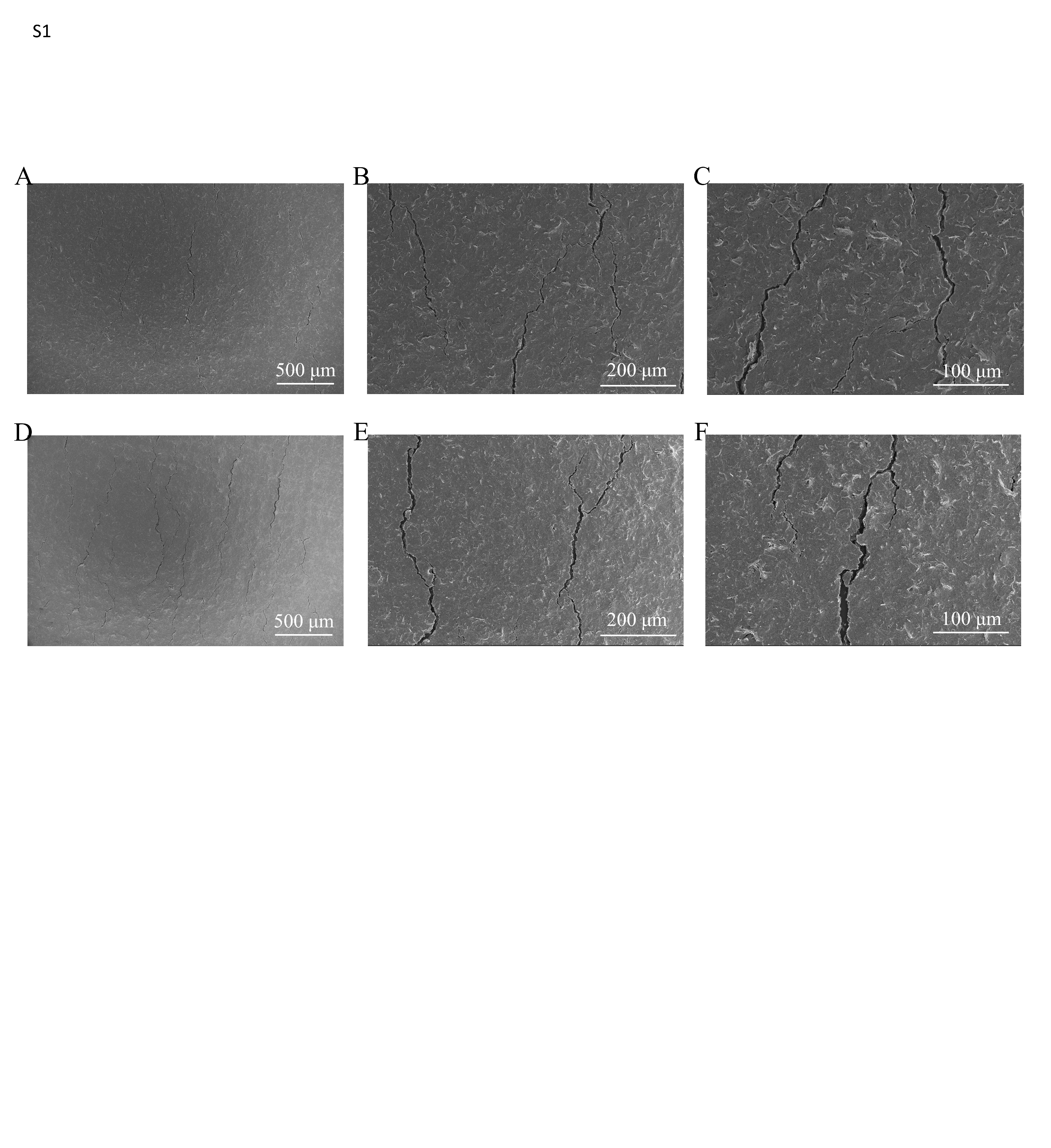


Fig. S7. SEM images of the microcracked conductive channels at different magnifications without strain (A-C) and with 5% strain (D-F). The scale bars are 500 μm for (A) and (D), 200 μm for (B) and (E), 100 μm for (C) and (F), respectively.


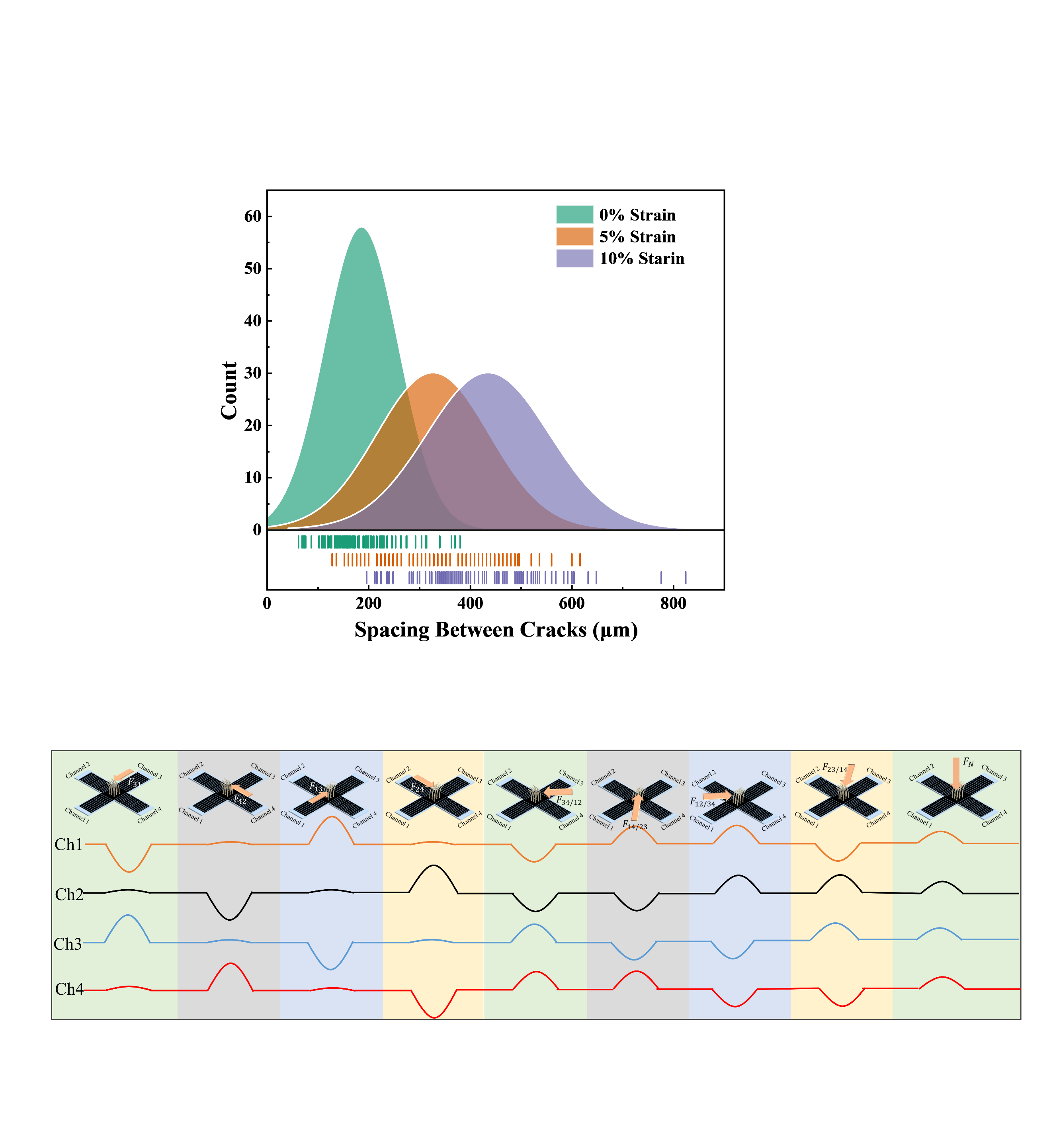


**Fig. S8.** **The statistic spacing between the corresponding cracks under different tensile strains.**


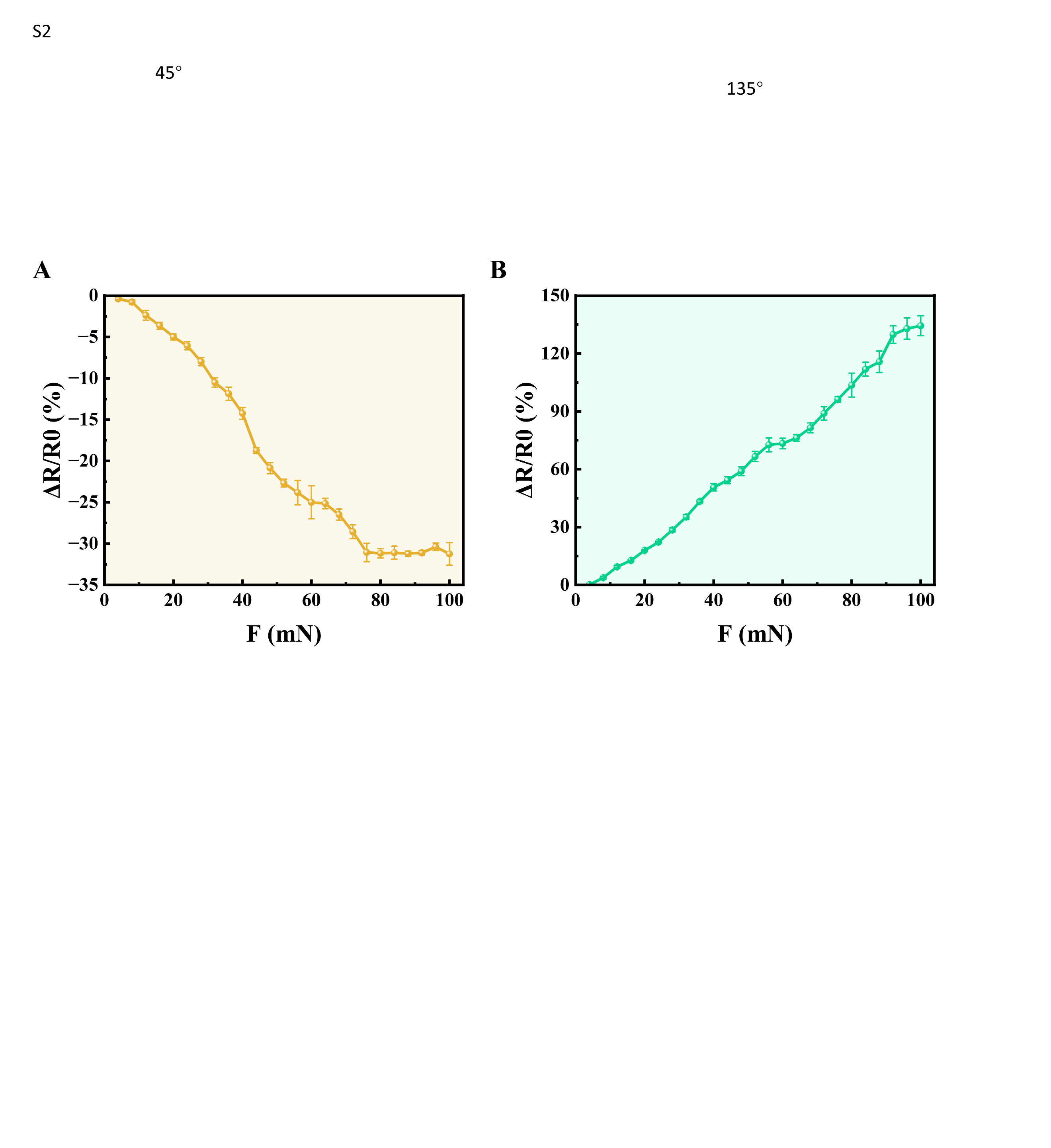


Fig. S9. Response behaviors of the microcracks channel 3 (A) and microcracks channel 1 (B) under an increasing shear force $F_{14/23}$.

**
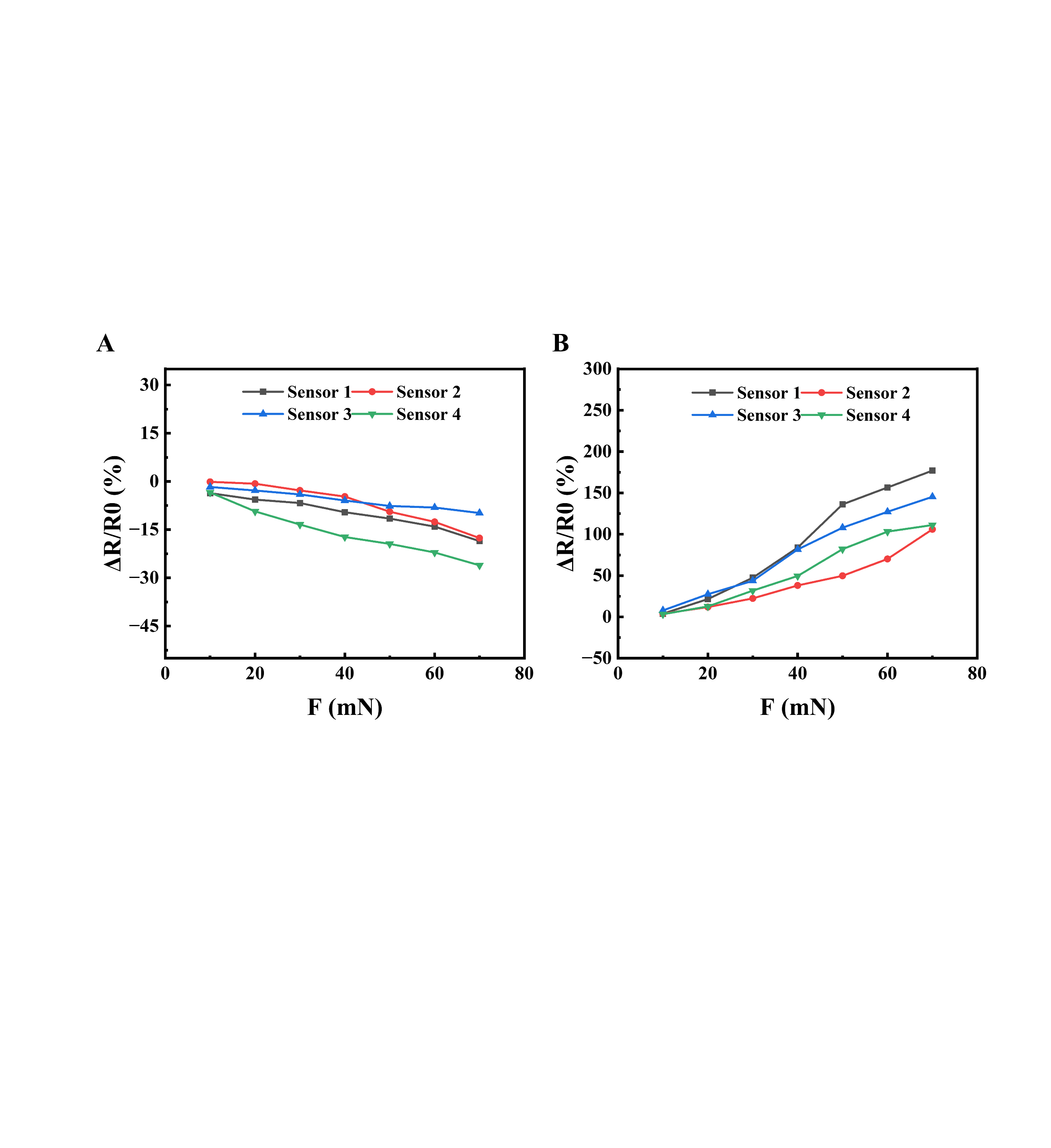
**

Fig. S10. Similar response behaviors of the four sensors under an increasing shear force $F_{13}$ and $F_{31}$

**
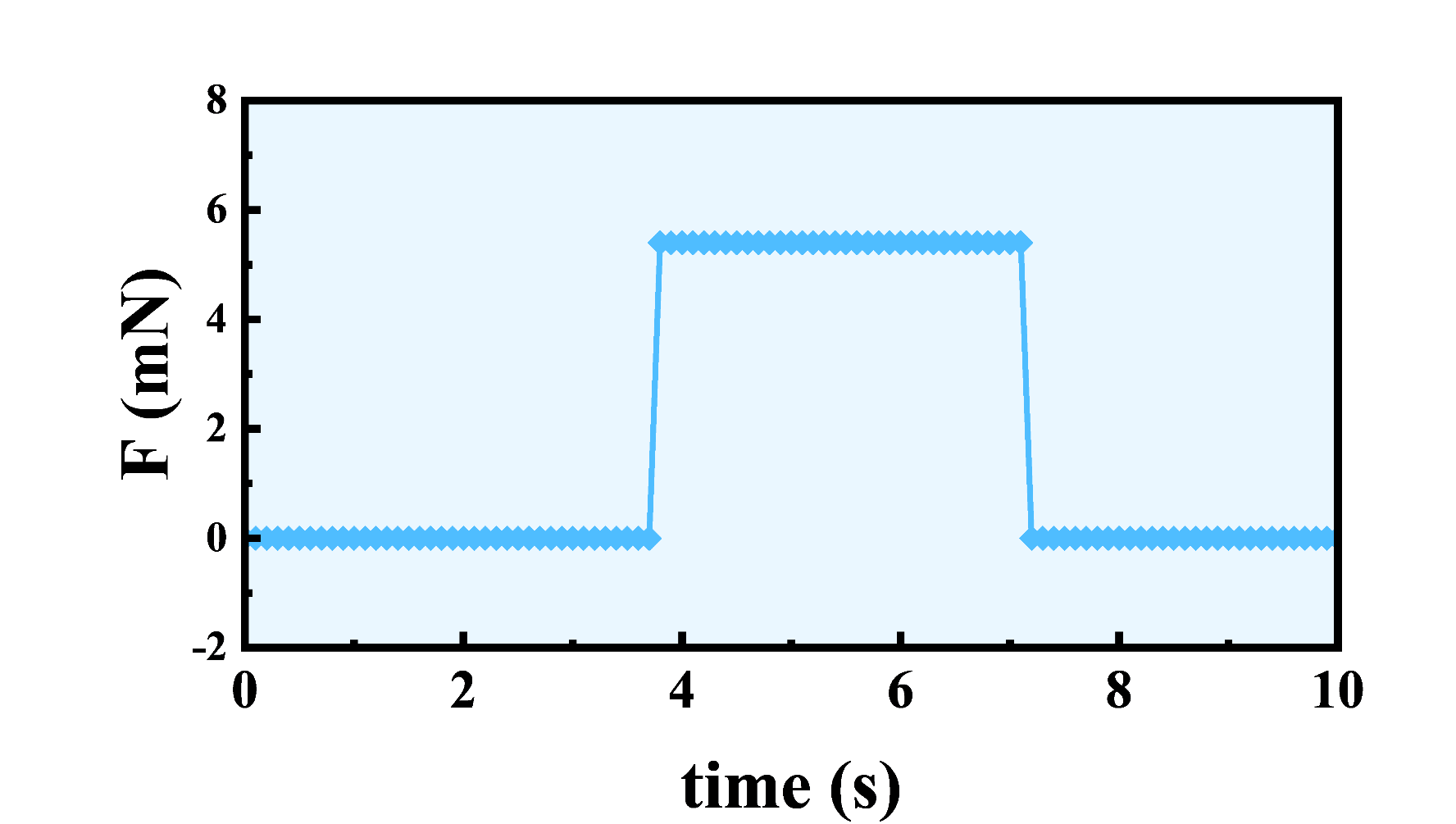
**

Fig. S11. A tiny shear force variation applied to tactile sensor to evaluate its lowest detection limit.

**
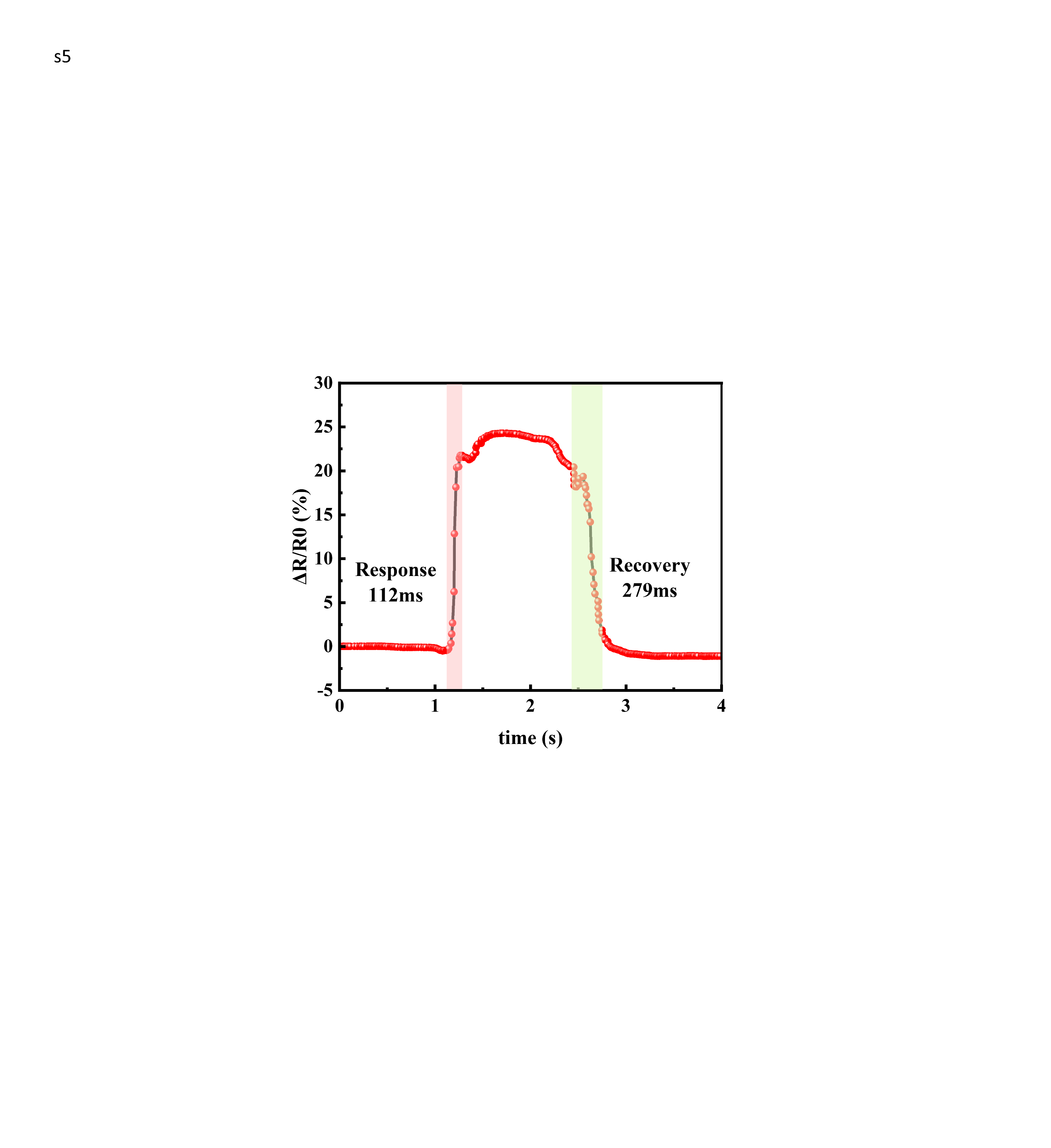
**

Fig. S12. Response time and recovery time of the tactile sensors.


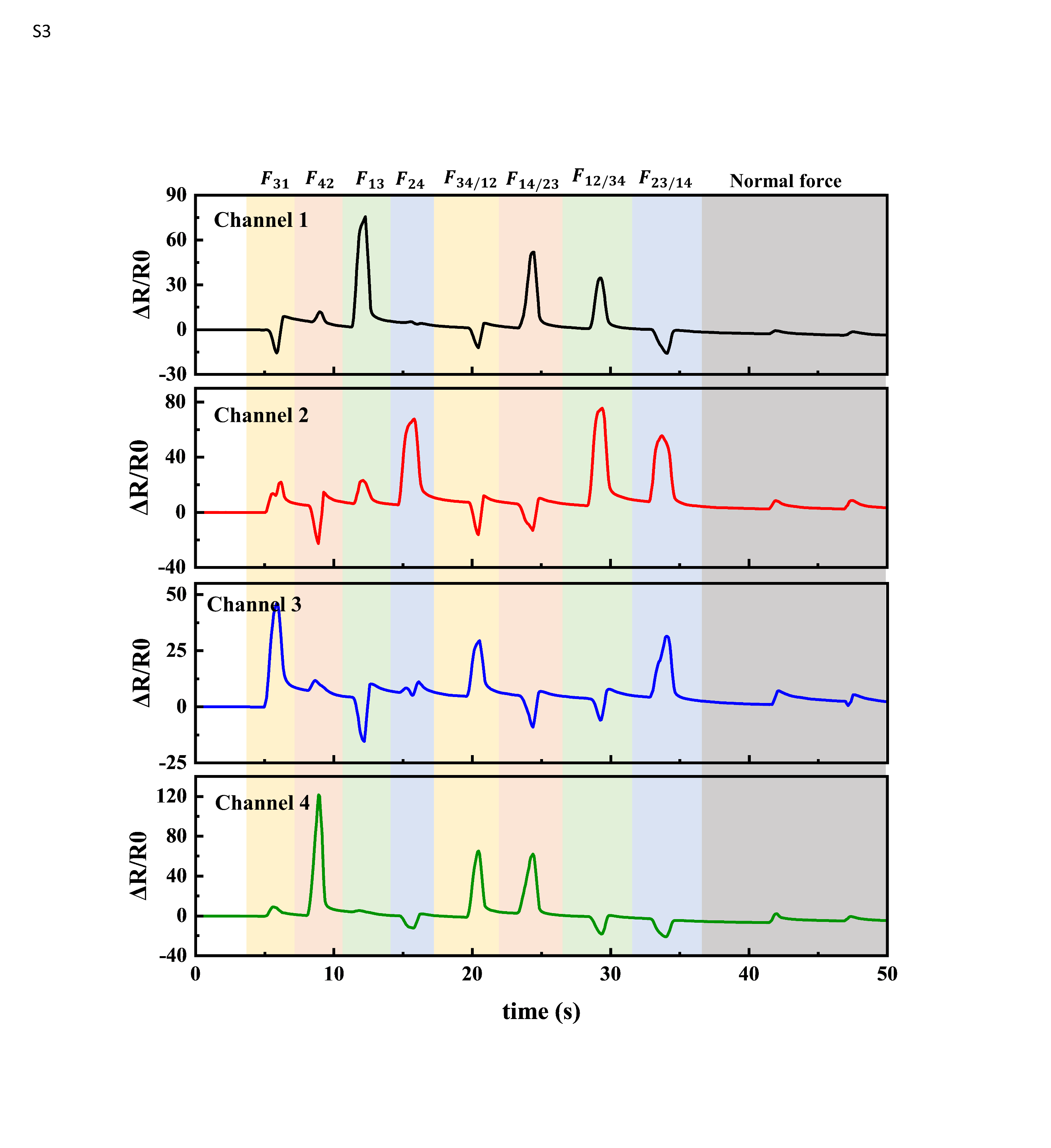


Fig. S13. Typical response behaviors of the four sensor channels when applying and removing shear forces of different directions and normal force.


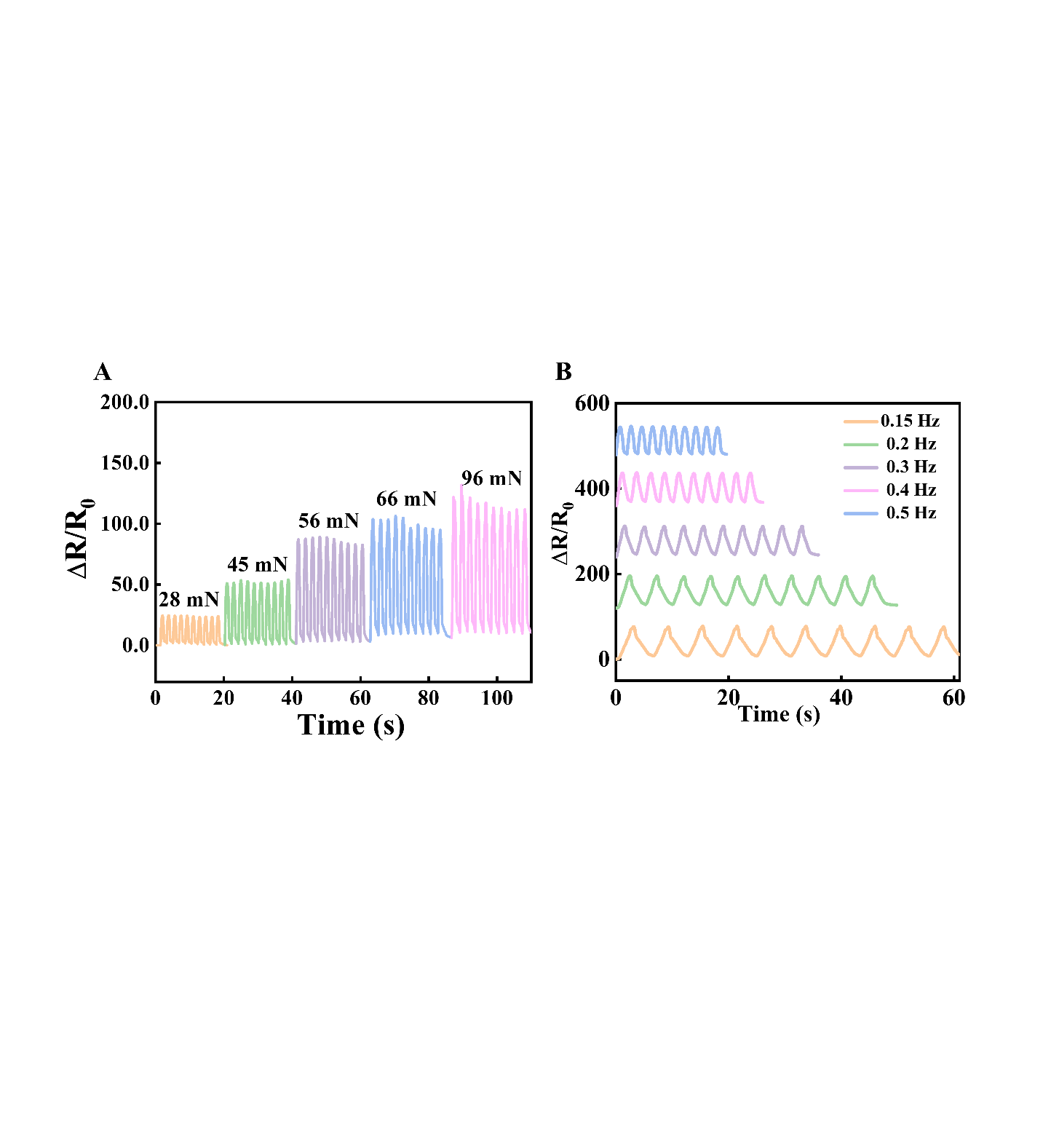


**Fig. S14.** **Responses of the sensor under different shear forces (A) and at different rates (B) in ten cycles test.**


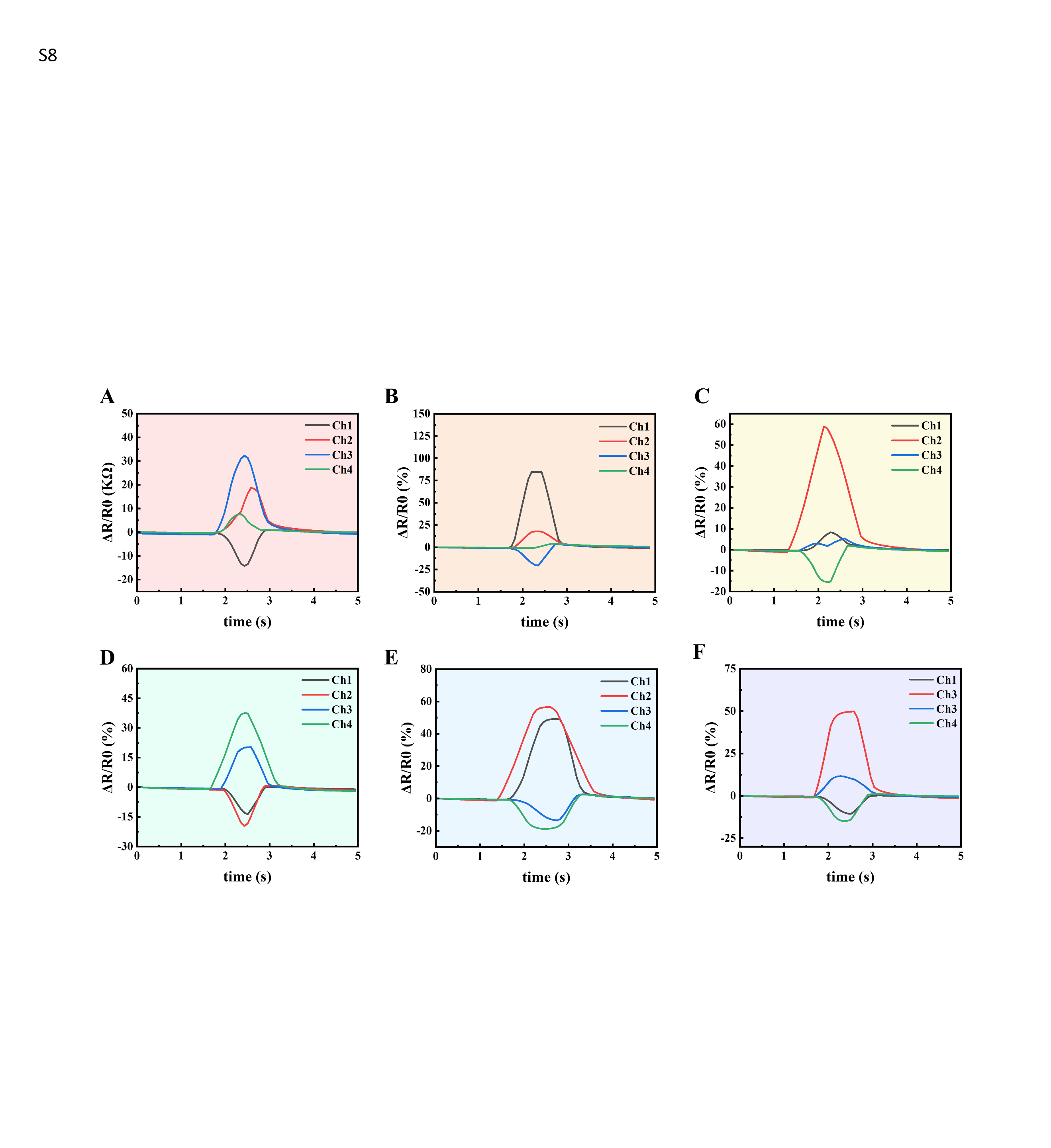


Fig. S15. Typical response behaviors of the four sensor channels under shear forces of different directions: $F_{31}$ (A), $F_{13}$ (B), $F_{24}$ (C), $F_{34/12}$ (D), $F_{12/34}$(E), $F_{23/14}$(F).


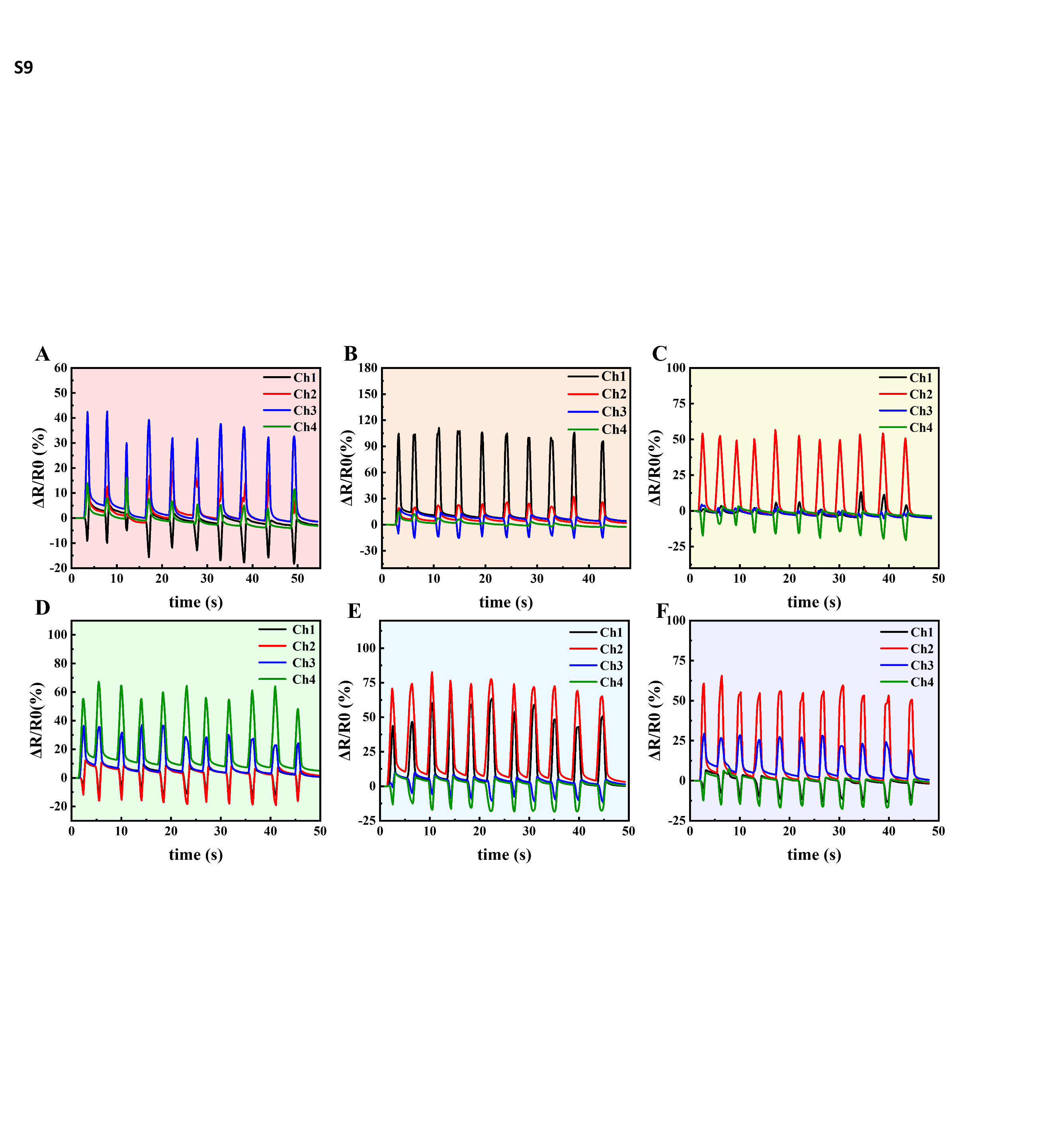


Fig. S16. Reliability test of the sensors when loading-unloading 10 cycles of shear force with different directions: $F_{31}$ (A), $F_{13}$ (B), $F_{24}$ (C), $F_{34/12}$ (D), $F_{12/34}$(E), $F_{23/14}$(F).


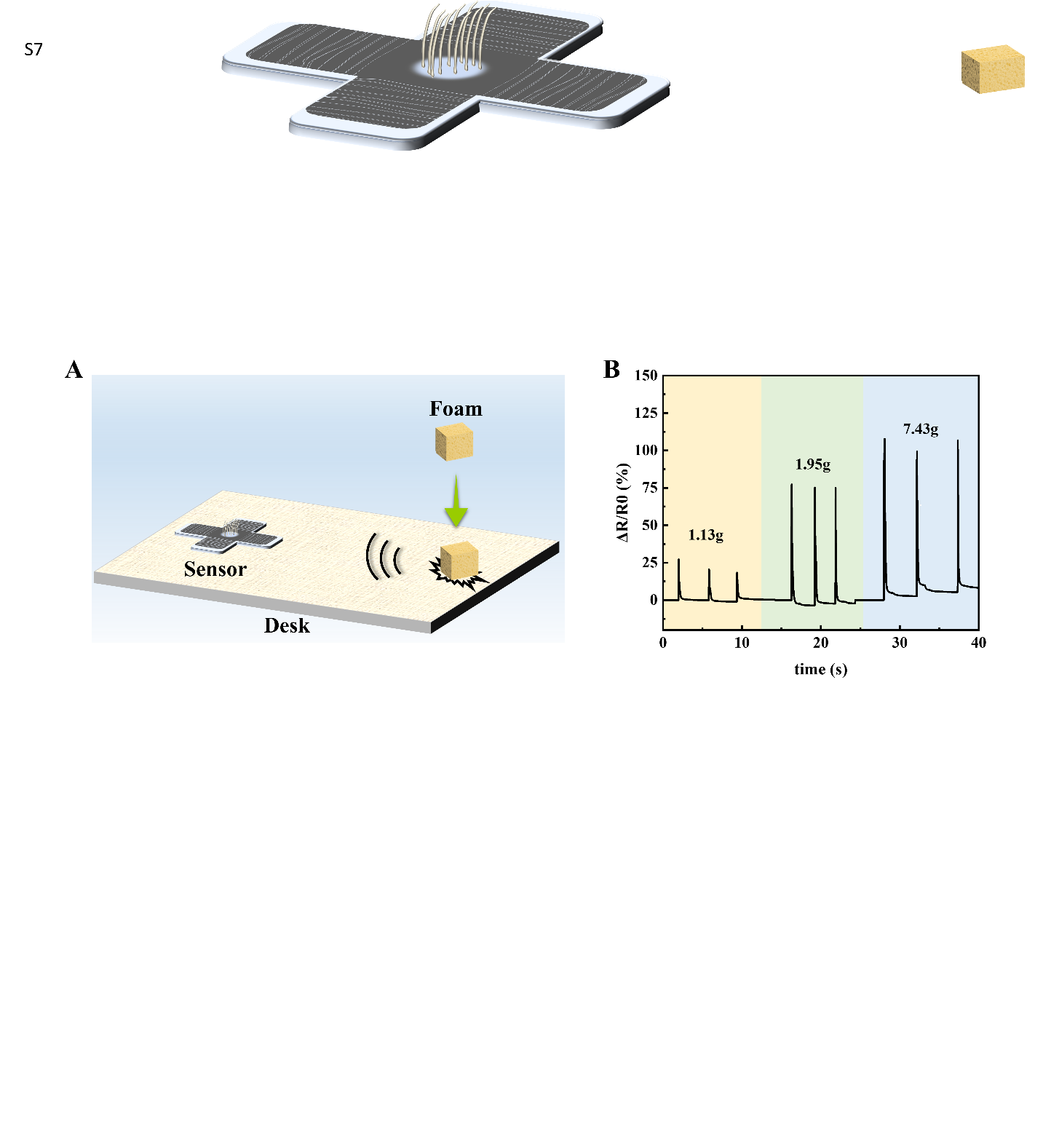


Fig. S17. Schematic diagram and response curve of the sensor for detecting different vibration intensity. (A) Objects of different weights fell on the desk from a height of 10 cm, causing the desk to vibrate. Horizontal distance between object and sensor is 30cm. (B) The tactile sensor showed different response amplitudes with objects of different weights fallen on the desk.

**Supplementary Note S3:**

The interaction between the conductive ink and the PDMS/PVA films is important for the sensing stability for tactile sensors. PDMS has the characteristics of high hydrophobicity and low surface energy, leading to the weak adhesion between PDMS and conductive carbon paste, which seriously affects the performance of the sensor. Therefore, it is necessary to modify the surface of PDMS to improve the hydrophilicity. The plasma reacts with the surface of the treated material to activate the surface of the polymer, improving its hydrophilicity and enhancing the interfacial interaction. However, after plasma treatment of PDMS, it was found that the hydrophilicity of modified PDMS would decrease with the passage of time. The sensing properties of the microcracked carbon ink channel are also affected. Considering that PVA has a good hydrophilicity, we deposited a layer of PVA on the surface of PDMS after plasma treatment, so as to simply and quickly produce a durable and hydrophilic PDMS surface for conductive ink^57,58^. Based on the advantages of PVA, there is a strong interaction between the conductive ink and PDMS/PVA, which also ensures the excellent stability of microcrack sensing film.
